# Strontium/magnesium-doped coralline hydroxyapatite for bone regeneration

**DOI:** 10.1093/rb/rbaf036

**Published:** 2025-05-21

**Authors:** Bixiu Chen, Liyan Zhang, Zhou Zhong, Chunyu Liu, Haobo Pan

**Affiliations:** Shenzhen Key Laboratory of Marine Biomedical Materials, CAS-HK Joint Lab of Biomaterials, Shenzhen Institutes of Advanced Technology, Chinese Academy of Sciences, Shenzhen 518055, P.R. China; Shenzhen Key Laboratory of Marine Biomedical Materials, CAS-HK Joint Lab of Biomaterials, Shenzhen Institutes of Advanced Technology, Chinese Academy of Sciences, Shenzhen 518055, P.R. China; Shenzhen Healthemes Biotechnology Co., Ltd, Shenzhen 518102, P.R. China; Shenzhen Key Laboratory of Marine Biomedical Materials, CAS-HK Joint Lab of Biomaterials, Shenzhen Institutes of Advanced Technology, Chinese Academy of Sciences, Shenzhen 518055, P.R. China; Shenzhen Key Laboratory of Marine Biomedical Materials, CAS-HK Joint Lab of Biomaterials, Shenzhen Institutes of Advanced Technology, Chinese Academy of Sciences, Shenzhen 518055, P.R. China; Shenzhen Healthemes Biotechnology Co., Ltd, Shenzhen 518102, P.R. China

**Keywords:** coral, strontium/magnesium-doped coralline hydroxyapatite, ion release, osteoinductivity, osteogenesis

## Abstract

The biocompatibility, osteoconductivity and porous structure of coral make it a popular material for bone regeneration. However, coral mismatches host bone degradation rates and lacks osteoinductivity. No prior research has investigated the physicochemical properties of strontium-doped coralline hydroxyapatite (Sr-CHA), magnesium-doped (Mg-CHA) and strontium- and magnesium-co-doped (Sr-Mg-CHA), especially their osteogenic mechanisms. This study synthesized CHA doped with osteoinductive elements (Sr, Mg and Sr-Mg) via a hydrothermal reaction to preserve 26.5–33.5% of the unconverted inner core of calcium carbonate (CaCO_3_). Under identical reaction circumstances, the Sr doping ratio in the Sr-CHA outperformed Mg in the Mg-CHA. In contrast, Sr and Mg mutually inhibit each other during co-doping in the Sr-Mg-CHA. The Sr-CHA nanorods on nanocluster spheres were the longest, while the Mg-CHA were the shortest, with the Sr-Mg-CHA occupying an intermediate length. The Sr-CHA, Mg-CHA and Sr-Mg-CHA exhibited 16 times the specific surface area and 14 times the pore volume of the coral and displayed better biocompatibility and expression levels of osteogenesis-related genes and proteins (e.g. ALP, Runx2, COL I, OCN and OPN) compared to coral *in vitro*, as well as improved osteogenesis than coral or Bio-Oss^®^  *in vivo*. With its optional Sr^2+^ release concentration and degradation rates and large specific surface area and pore volume, the Sr-CHA performs the best. This study improved bone tissue engineering and regenerative medicine by enhancing the understanding of doped CHA and revealing new ways to overcome bone repair material problems.

## Graphical Abstract

**Graphical Abstract:**
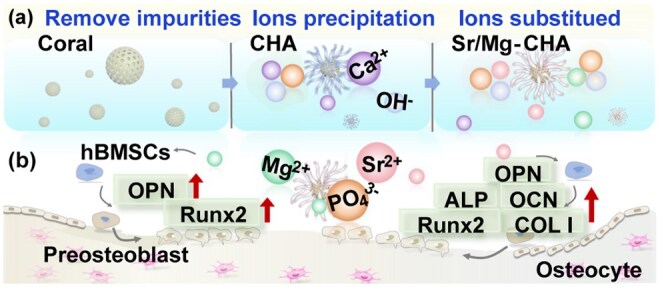

## Introduction

Worldwide, bone injuries resulting from trauma, infections, tumors, congenital abnormalities or aging total hundreds of millions of cases per year, including around 150–200 million fractures and about 8.9 million osteoporotic fractures [1]. These bone injuries cost hundreds of billions to treat [2]. Although autologous bone grafting is the gold standard for bone repair, donor scarcity, long surgery times and donor site infections limit its utilization. Allografts and xenografts reduce donor scarcity but risk immunological rejection and disease transmission [3, 4]. Bio-Oss^®^, a xenograft formed from deproteinized bovine bone mineral granules containing hydroxyapatite (HA, Ca_10_(PO_4_)_6_(OH)_2_), known for its non-antigenic properties, porous architecture, superior biocompatibility and osteoconductivity, has been used in dental implants and maxillofacial and periodontal bone defects alongside barrier membranes for decades [5–7]. However, recent clinical results show that Bio-Oss^®^'s high pricing, inconsistent degradation rates and low osteoinductivity limit its use in bone regeneration [8, 9].

As the world population ages, bone injuries and their economic burden will rise. Thus, effective and affordable bone graft materials with low disease transmission and tunable biophysical and biochemical properties have garnered significant attention [10, 11]. The optimal bone graft material should have superior biocompatibility, degradation rates, porous structure, osteoconductivity and osteoinductivity to drive endogenous progenitor or stem cells to the injury site and help heal damaged bone tissue [12].

Coral, composed of 95–99% CaCO_3_, has a cancellous bone-like structure, a pore size of 100–500 µm, a porosity of 40–73%, and a compressive strength of 2.62–12.06 MPa. A growing body of literature has focused on coral due to its biocompatibility and osteoconductivity, which make it a promising scaffold for bone tissue engineering [13–19]. Despite the existence of approximately 7000 coral species, surveys such as that conducted by Wu *et al*. indicated that Goniopora sp. is among the most promising sources of scaffolds for bone tissue engineering, attributed to its high porosity (73%), permeability and mechanical properties similar to cancellous bone [18]. However, the performance of coral is limited by the fast degradation in living organisms, which inadequately supports new bone formation and exhibits insufficient osteoinductivity, leading to suboptimal osteogenesis [15, 20, 21]. To overcome these limitations, several researchers have utilized the hydrothermal method to fully convert coral into HA, a material with a mineral composition akin to human bone that maintains coral's porosity structure and biocompatibility. Nonetheless, HA lacks osteoinductivity and degrades too slowly to form new bone [22–25]. Recent research suggests that partial coral conversion to HA with a CaCO_3_ core can improve biodegradability [23].

Ion doping, the process of incorporating specific metal ions into biomaterials like HA, significantly enhances osteogenesis by influencing bone regeneration through multiple mechanisms [26]. This strategy is particularly promising as it activates key signaling pathways (e.g. Wnt/β-catenin pathway), which stimulates the differentiation of osteoblasts, the bone-forming cells [27]. Additionally, ion doping enhances the bioactivity of biomaterials, promoting the adhesion and proliferation of osteogenic cells, and modulates the local inflammatory response to create a more favorable environment for bone tissue repair [27, 28].

Previous studies show a strong and consistent link between Sr^2+^ or Mg^2+^ and osteogenesis. Sr^2+^ has a dual function in bone regeneration by enhancing osteoblast proliferation and decreasing osteoclast activity [26, 27, 29–31]. Mg^2+^ can increase osteoblast proliferation and differentiation, promoting bone formation and development [26, 32, 33]. The integration of these metal ions into HA has been shown to significantly enhance osteogenesis [26]. To better mimic biological apatite, several researchers have synthesized Sr- or Mg-doped HA [14, 27, 34]. As summarized in Supplementary Table S1, current HA-based biomaterials are devoid of long-term stability studies of materials in dynamic physiological environments (e.g. degradation rate and ion release behavior), and there is inadequate validation of *in vitro* biological properties and *in vivo* biocompatibility of the materials, especially long-term animal model evaluations.

Strontium-doped coralline hydroxyapatite (Sr-CHA) was first addressed and analyzed by Liu *et al*., who found that doping 6–14% Sr to CHA can boost MC3T3-E1 cell proliferation and osteogenesis *in vivo* [14]. Despite convincing evidence that Sr^2+^ can increase CHA bioactivity, prior studies have not been studied human bone marrow mesenchymal stromal cells (hBMSCs), which are commonly utilized in tissue engineering due to their secretion of various bioactive molecules that affect angiogenesis, immunomodulation and osteogenesis. Moreover, the physicochemical properties and mechanisms of strontium/magnesium-doped coralline hydroxyapatite (Sr/Mg-CHA), a mixture of the Sr-CHA, Mg-CHA and Sr-Mg-CHA, have not been well examined in bone regeneration.

Thus, this study aims to address coral's inconsistent degradation rates and low osteoinductivity as a bone regeneration scaffold. This study synthesized the Sr-CHA, Mg-CHA and Sr-Mg-CHA with an unconverted CaCO_3_ core via a hydrothermal method and analyzed their phase composition, functional groups, surface characteristics, chemical composition, morphology, degradation and ion release behavior. These materials were also evaluated *in vitro* for biocompatibility, osteogenesis-related gene and protein expression, and *in vivo* bone regeneration compared to Bio-Oss^®^ in a rat femoral condyle defect model to determine their osteogenic activity and mechanism. This study aspires to advance bone tissue engineering by developing degradable and osteoinductive bone repair materials for orthopedics and dentistry.

## Materials and methods

### Preparation of the Sr/Mg-CHA

The study used Goniopora sp. from the South China Sea near Sansha, China, along with diammonium hydrogen phosphate ((NH_4_)_2_HPO_4_, AR, Sinopharm Chemical Reagent, China); strontium nitrate (Sr(NO_3_)_2_, AR, Shanghai Lingfeng, China); magnesium nitrates hexahydrates (Mg(NO_3_)_2_·6H_2_O, AR, Shanghai Lingfeng, China); and ammonium hydroxide (NH_3_·H_2_O, NH_3_ 25–28%, Shanghai Lingfeng, China). The coral was soaked in 5 wt% sodium hypochlorite at room temperature for 4 days to remove pollutants and proteins, then rinsed with distilled water, ultrasonic cleaned and dried. The coral was pulverized with a 28 000 rpm grinding machine, filtered through a 1-mm mesh, and then through a 0.25-mm mesh. CHA, an intermediate that has a CaCO_3_ core and HA on the surface, was produced by combining 80 mL of 2 mol/L (NH_4_)_2_HPO_4_ solution with 4 g of coral, aging under magnetic stirring for 12 h, and reacting in a hydrothermal autoclave at 180°C and 0.5 MPa for 24 h at a pH of approximately 10. It was rinsed with distilled water, ultrasonic cleaned, and dried. After that, 4 g of the intermediate was added to 80 mL of 0.67 mol/L Sr(NO_3_)_2_, Mg(NO_3_)_2_, and a mixed solution of the two. After 12 h of magnetic stirring, the reaction continued for 24 h. The Sr-CHA, Mg-CHA and Sr-Mg-CHA were successfully generated following cleaning, rinsing with deionized water, distilled water, ultrasonic cleaning and drying. The reaction equations for the conversion of coral to CHA and for Sr/Mg-CHA are described in Equations 1 and 2, respectively.

(1) $$10{\text{CaCO}}_{3}+6({\text{NH}}_{4})2{\text{HPO}}_{4}\to {\text{Ca}}_{10}({\text{PO}}_{4})6(\text{OH}{)}_{2}+10{\text{CO}}_{2}+12{\text{NH}}_{3}+8{\text{H}}_{2}\text{O}$$

 

(2) $$\begin{array}{l} {\text{Ca}}_{10-x-y}{\left({\text{PO}}_{4}\right)}_{6}(\text{OH}{)}_{2}+x\text{Sr}{\left({\text{NO}}_{3}\right)}_{2}+y{\text{Mg}({\text{NO}}_{3})}_{2}\;\\ \to {\;\text{Ca}}_{10-x-y}\text{Sr}x\text{Mg}y({\text{PO}}_{4})6{\left(\text{OH}\right)}_{2}+(x+y)\text{Ca}{\left({\text{NO}}_{3}\right)}_{2} \end{array}$$

where *x* and *y* represent the moles of Sr and Mg doping, respectively.

### Characterization of the Sr/Mg-CHA

Multiple analytical methods were used to characterize the physicochemical properties of the coral, Sr-CHA, Mg-CHA, and Sr-Mg-CHA. X-ray diffraction (XRD, Ultima4, Rigaku, Japan) was used to analyze phase composition using Cu-Kα radiation at 40 kV and 100 mA, with diffraction angles from 5° to 95° in step scan mode (2θ = 0.02°). Functional groups were determined using Fourier transform infrared spectroscopy (FTIR, Bruker ALPHA, USA) with 4 cm^−1^ spectrum resolution in the 400–4000 cm^−1^ range. Morphology and semi-quantitative elemental composition were analyzed using a scanning electron microscope (SEM, Sigma 300, Zeiss, Germany) and energy-dispersive X-ray spectroscopy (EDX, Quantax XFlash SDD 6, Bruker, USA). The samples' quantitative elemental composition was determined using Inductively Coupled Plasma Mass Spectrometry (ICP-OES, Agilent 720, Agilent, USA) to determine the HA conversion ratio and Sr- or Mg-doped ratios. Automatic Specific Surface Area and Pore Size Analyzer (ASAP 2460/2020, Mark, USA) measured pore volume and specific surface area.

### Conversion ratios of HA and doping ratios of Sr or Mg from ICP analysis

We defined that the conversion ratio is the ratio of moles of Ca in HA of CHA to total Ca in CHA. Due to the absence of elemental phosphorus (P) in coral, the mass percentage of P can be used to calculate HA moles of CHA. The 10:6 Ca/P molar ratio was used to calculate HA's Ca moles. The mass percentage of Ca in CHA determined the total moles of Ca. The equation is described in (3).

Doping ratios were the proportion of Ca substituted by Sr and Mg in the Sr-CHA, Mg-CHA and Sr-Mg-CHA. This study used the aforementioned method to calculate doping moles by removing the coral's Sr and Mg mass percentages. Then, the moles of Ca and Sr, Ca and Mg, and Ca and Sr-Mg doped were calculated by subtracting the coral's mass percentage of P from the Sr-CHA, Mg-CHA and Sr-Mg-CHA. Doping ratio equations are in Equation 4.

(3) $${\text{Conversion}\;\text{ratio}}_{x/y-\text{CHA}}=\frac{n_{\text{Ca},\;\text{HA}(x/y-\text{CHA})}}{n_{\text{Ca},\;x/y-\text{CHA}}}=\left(\frac{(m\times W_{P})\;÷\;M_{P}}{(m\times W_{\mathrm{Ca}})\;÷\;M_{\mathrm{Ca}}}\times \frac{10}{6}\right)\times 100\%$$

 

(4) $$\begin{array}{c} {\text{Doping}\;\text{ratio}}_{x^{2+}(y^{2+}),\;x/y-\text{CHA}}=\frac{n_{x(y),\;x/y-\text{CHA}}-n_{x(y),\;\text{Coral}}\;}{n_{\text{Ca},\;\text{HA}(x/y-\text{CHA})}}\;\;\\ =\left(\frac{n_{x(y),\;x/y-\text{CHA}}-n_{x(y),\;\text{Coral}}}{(m\times W_{P})÷M_{P}}\times \frac{6}{10}\right)\times 100\% \end{array}$$

where *x* = Sr, *y* = Mg and *x*/*y* indicates Sr, Mg or both. The *n*_Ca, HA(__*x*__/__*y*__-CHA)_ denotes the moles of Ca in HA converted from coral in *x*/*y*-CHA. The *n*_Ca,_ _*x*__/__*y*__-CHA_ denotes the moles of Ca in *x*/*y*-CHA. The *m* denotes the mass of the sample. The *n_x_*_(__*y*__),_ _*x*__/__*y*__-CHA_ represents the moles of dopant atoms in the Sr-CHA, Mg-CHA and Sr-Mg-CHA. The *n_x_*_(__*y*__), Coral_ denotes the moles of the *x* or *y* in the coral. The *W*_P_ signifies the percent mass of P, excluding the P present in the coral. The *W*_Ca_ represents the mass percentage of Ca in the samples. The *M*_Ca_ is the molar mass of Ca. The *M*_P_ is the molar mass of P.

### *In vitro* degradation and ion release behavior of the Sr/Mg-CHA

For degradation and ion release tests, 2 g of the coral, Sr-CHA, Mg-CHA and Sr-Mg-CHA were distributed in 10 mL of Tris-HCl buffer (0.1 M, pH 7.4) and PBS, respectively. The suspensions were shaken at 37°C to replicate dynamic conditions for 28 days. Remove 1 mL of liquid from PBS after 1, 3, 7, 14 and 28 days and replenish with fresh PBS. The Ca^2+^, Mg^2+^ and Sr^2+^ concentrations were measured by ICP-OES. After filtering and drying, the samples were weighed, and the immersion solutions were collected and refilled with 10 mL of fresh buffer after 7, 14, 21 and 28 days. Weight loss was calculated using Equation 5.

(5) $$\text{Weight}\;\text{loss}=\frac{M_{0}-M_{t}}{M_{0}}\times 100\%$$

where *M*_0_ is the mass of the samples before immersion and *M*_t_ is the mass of the samples at the test time.

### *In vitro* cellular response of hBMSCs to Sr/Mg-CHA

#### Cell culture and extract preparation

The hBMSCs (PCS-500-012™, ATCC^®^, USA) were cultured in alpha minimum essential medium (α-MEM, SH30265.01, HyClone, China) supplemented with 10% fetal bovine serum (MK1128-500, MIKX, China) and 1% penicillin-streptomycin solution (SV30010, Hyclone, China) in a cell culture incubator environment (37°C, 5% CO_2_, and saturated humidity). The cells were digested with a 0.25% trypsin EDTA solution (SH30042.01, HyClone, China).

The extracts of the coral, Sr-CHA, Mg-CHA and Sr-Mg-CHA were prepared by immersing them in the α-MEM medium at a concentration of 0.2 g/mL by ISO 10993-12:2021. To obtain sterile extracts, the samples were soaked in α-MEM medium and filtered through 0.22-µm sterile filters (MK10322, MIKX, China) after 24 h of continuous agitation at 37°C. The extracts were later utilized for *in vitro* cellular assays to evaluate biocompatibility and cellular responsiveness. The cell culture medium without materials under the same extraction conditions was used as the control.

Detailed *in vitro* adhesion and proliferation, ALP and ARS osteogenic-induced differentiation staining, osteogenesis-related genes and protein expression are shown in Supplementary Material S2.1.

#### Hemolysis rates

A hemolysis test was conducted according to the ISO 10993-4:2017. New Zealand White rabbits aged 8 weeks had blood collected in tubes containing an anticoagulant (XK002010, Shenzhen Huashi, China), namely 0.109 mol/L sodium citrate. The procedure of hemolysis test is detailed in Supplementary Material S2.2. A hemolysis rate <5% suggests being hemocompatible. A hemolysis rate ≥5% means having a hemolytic effect. The hemolysis rate is calculated by Equation 6.

(6) $$\text{Hemolysis}\;\text{rate}=\frac{A-A_{2}}{A_{1}-A_{2}}\times 100\%$$

where *A* is the absorbance of the sample, *A*_1_ is the absorbance of the positive control and *A*_2_ is the absorbance of the negative control.

### *In vivo* assessment of the Sr/Mg-CHA

#### Implant selection and surgical procedure

All animal operations and experiments were conducted in accordance with the guidelines for the care and use of laboratory animals and were approved by the Institutional Animal Care and Use Committee (IACUC) of the Shenzhen Institutes of Advanced Technology, Chinese Academy of Sciences (Approval Number: SIAT-IACUC-240328-YYS-CX-A2541). Male Sprague–Dawley rats aged 6–8 weeks were used to create a cylindrical lateral femoral condyle bone defect model. A drill bit drilled 2.5-mm holes in rats' bilateral femorals during sterile surgery. Each hole was implanted with 2 g of the Bio-Oss^®^ (0.25–1 mm, Geislich AG, Switzerland), coral, Sr-CHA, Mg-CHA and Sr-Mg-CHA (*n* = 4). The animals were necropsied at 4 and 8 weeks post-surgery, and the femoral samples were submerged in 4% paraformaldehyde at room temperature for 2 weeks.

#### In vivo osteogenic properties

Samples were scanned using Micro-CT (µCT 100, Scanco, Switzerland) on the reconstructed femur after 4% paraformaldehyde fixation to assess new bone growth in the defect area. Three-dimensional reconstructions were performed using CTAn and CTvox software with a 2.5-mm diameter and implant area as the ROI (region of interest) after the scan. Bone mineral density (BMD), bone volume fraction (BV/TV) and trabecular thickness (Tb.Th) were quantified in ROIs to assess osteogenesis.

#### Histomorphometric evaluation

After micro-CT scanning, samples were decalcified in an alkaline solution containing 0.67 mol/L disodium ethylenediaminetetraacetic acid (110702062, Bikeman Bio, China) at pH 7–8, embedded in paraffin, sectioned and stained according to standard histological protocols. Detailed experimental protocols and reagent specifications are provided in the Supplementary Material S2.3. The sections were stained with hematoxylin and eosin (HE) to evaluate overall histomorphology and with Masson's trichrome (Masson) to visualize collagen fibers. Pathological large tissue whole section scan equipment (Pannoramic MIDI, 3DHISTECH Ltd, Hungary) and CaseViewer software were used to image bone growth *in vivo*.

### Data analysis

The one-way analysis of variance (ANOVA) and two-way ANOVA (GraphPad Prism 10.0) were used to assess the statistical differences, followed by Tukey's multiple comparisons test. Significant differences in quantitative analyses of cellular proliferation and bone regeneration at defect sites were analyzed by two-way ANOVA. Hemolysis, osteogenesis-related genes and proteins expression were used with one-way ANOVA to assess significant differences. ANOVA was preceded by Shapiro–Wilk test. The value of *P < 0.05, **P < 0.01, ***P < 0.001 and ****P < 0.0001 indicates significant differences. The value of *P *> 0.05 (ns) means no significant difference.

## Results

### Preparation and characterization of the Sr/Mg-CHA

**Figure 1.**
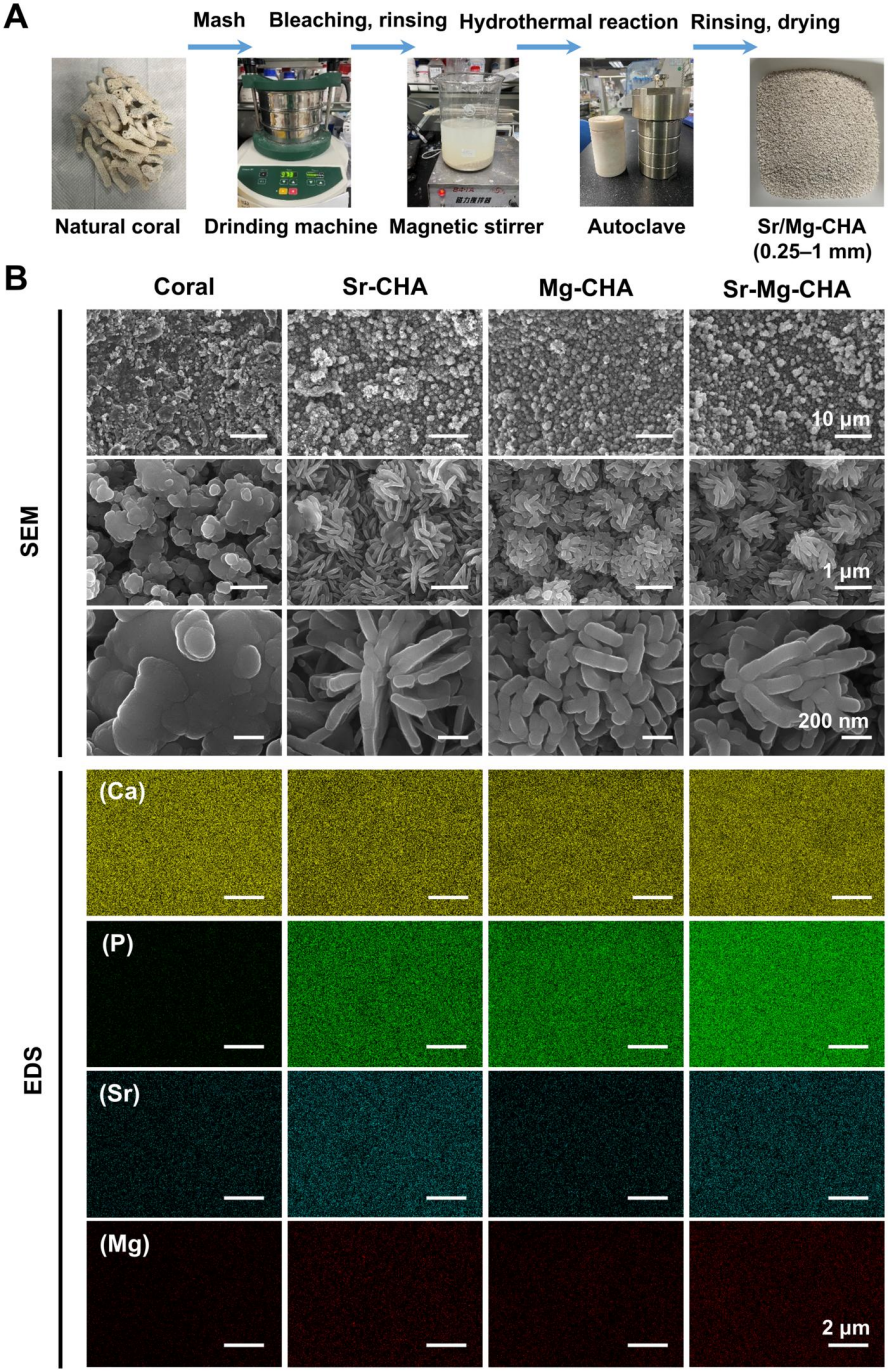
Materials preparation, morphology, and composition of the coral, Sr-CHA, Mg-CHA and Sr-Mg-CHA. (**A**) Preparation process. (**B**) SEM images and elemental mapping.

A two-step hydrothermal reaction produced the Sr-CHA, Mg-CHA and Sr-Mg-CHA with a CaCO_3_ core. The preparation process was as follows: after removing impurities, the coral was crushed and sieved into 0.25–1 mm. CHA was synthesized using magnetic stirring and hydrothermal reaction. After aging with Sr(NO_3_)_2_ or Mg(NO_3_)_2_ solutions, the CHA was hydrothermally reacted. Figure 1A shows the final Sr/Mg-CHA after washing and drying.

#### Scanning electron microscopy with energy-dispersive X-ray spectroscopy

Figure 1B illustrates the morphology and elemental distribution of the coral, Sr-CHA, Mg-CHA and Sr-Mg-CHA. The coral's surface exhibited a systematic arrangement of lamellar and granular crystals. EDX examination confirmed that its primary composition consisted of Ca and minor Sr and Mg. Following hydrothermal reaction, the Sr-CHA, Mg-CHA and Sr-Mg-CHA groups had 1-µm nanocluster spheres with high P content, indicating successful conversion of coral into CHA. All samples had Ca/P molar ratios above 1.67 (Table 1). Nanorod length statistics are shown in Supplementary Figure S2. With Sr dispersion on the surface, Sr-CHA has nanocluster spheres and nanorods around 480 ± 85.9 nm. Compared to the Sr-CHA, the Mg-CHA had more surface defects, including nanorods on 265.5 ± 41.2 nm aggregated spheres and Mg-enriched surfaces. In the Sr-Mg-CHA, the surface morphology was more unusual. However, the nanorods changed to 394.3 ± 64.8 nm, and Mg and Sr accumulated on the surface. Figure 1B shows that hydrothermal reaction can change coral morphology from lamellar and granular nanocrystals to nanorods. Mg doping made nanorods irregular and shorter, while Sr doping did the opposite. Sr-Mg co-doping increased the material's irregularity, although the nanorods were shorter than Sr-doped nanorods alone.

**Table 1.** The elemental composition of the coral, Sr-CHA, Mg-CHA, and Sr-Mg-CHA from EDX

Samples	Ca (at.%)	P (at.%)	Sr (at.%)	Mg (at.%)	Ca/P	(Ca + Sr/Mg)/P^a^
Coral	20.76	0	0.20	0.09	—	—
Sr-CHA	15.84	9.54	1.03	0.11	1.75	1.77
Mg-CHA	15.82	9.13	0.16	0.67	1.73	1.80
Sr-Mg-CHA	17.56	10.56	0.90	0.47	1.76	1.77

a The coral's Sr and Mg proportion is not included. (Ca + Sr/Mg)/P combines (Ca + Sr)/P in Sr-CHA, (Ca + Mg)/P in Mg-CHA and (Ca + Sr + Mg)/P in Sr-Mg-CHA.

#### X-ray diffraction

**Figure 2.**
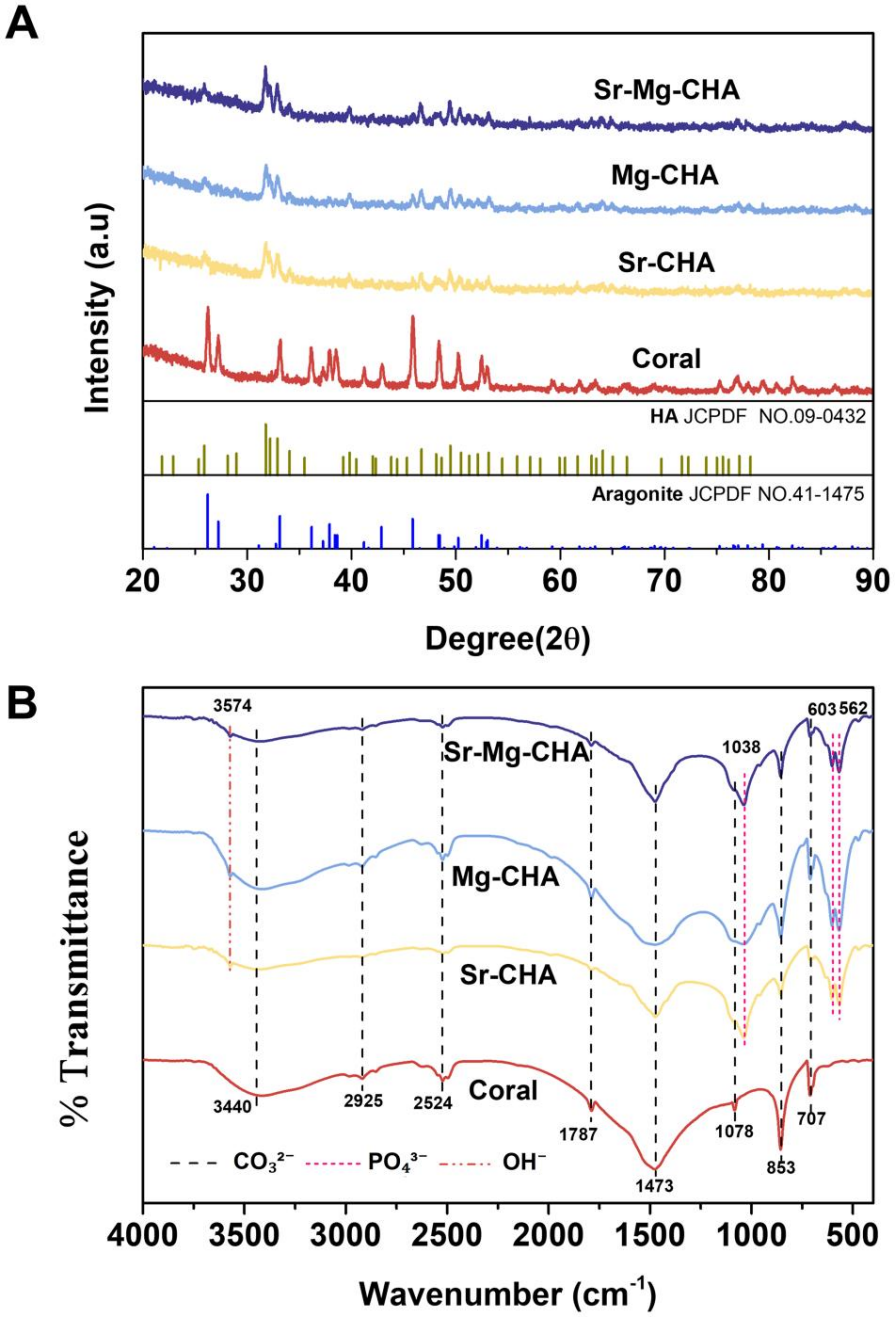
Phase composition and functional groups of the coral, CHA, Sr-CHA, Mg-CHA and Sr-Mg-CHA. (**A**) XRD patterns. (**B**) FTIR spectra.

Figure 2A shows the XRD patterns of the coral, CHA, Sr-CHA, Mg-CHA and Sr-Mg-CHA. The primary crystal phase of the coral is aragonite CaCO_3_, exhibiting diffraction peaks at 26.2°, 27.2°, 33.1°, 36.2°, 37.8°, 42.8°, 45.8° and 48.4°, in accordance with JCPDF No. 41-1475. The Sr-CHA, Mg-CHA and Sr-Mg-CHA diffraction peaks were observed at 25.9°, 31.7°, 32.2°, 32.9°, 34°, 39.8°, 46.7° and 49.5°, aligning with the characteristic peak positions on the HA standard card JCPDF No. 09-0432, along with a few peak positions of CaCO_3_. The findings demonstrate that the coral has partially converted to HA, and the Sr-CHA, Mg-CHA and Sr-Mg-CHA did not significantly change the diffraction peaks, indicating that these dopant elements did not meaningfully affect the samples' crystal structure.

#### Fourier transform infrared spectroscopy

Figure 2B shows that the FTIR spectrum of the coral is characterized by vibrational absorption peaks at 707 cm^−1^ (ν_4_, out-of-plane bending), 853 cm^−1^ (ν_2_, in-plane bending), 1078 cm^−1^ (ν_1_, symmetric stretching) and 1473 cm^−1^ (ν_3_, asymmetric stretching), which are linked with the carbonate ion (CO_3_^2−^) functional group of CaCO_3_ in aragonite. After hydrothermal reaction, the Sr-CHA, Mg-CHA and Sr-Mg-CHA displayed asymmetric stretching vibrational absorption peaks of the hydroxyl group (OH^−^) at 3574 cm^−1^ (ν_3_), bending vibrational absorption of phosphate ion (PO_4_³^−^) at 562 cm^−1^ (ν_4_) and 603 cm^−1^ (ν_4_), and asymmetric stretching vibrational absorption peaks of PO_4_³^−^ at 1038 cm^−1^ (ν_3_), resembling typical HA. The Sr-CHA, Mg-CHA and Sr-Mg-CHA also showed vibrational absorption peaks connected to the CO_3_^2−^ in CaCO_3_ of aragonite, supporting the XRD results (Figure 2A) and having nearly comparable FTIR spectra and functional group characteristics. As CaCO_3_ decomposes at 600–800°C and HA at 800–1200°C, further thermogravimetric analysis in Supplementary Figure S1 reveals that coral lost mass in stages: 2–3% from room temperature to 300°C (water evaporation) and another 2% from 300 to 650°C (lattice water and organics). At 650–830°C, CaCO_3_ decomposes, leaving 54% mass. While CHA exhibited a similar mass loss trend up to 830°C but retained 70% at 1000°C, indicating unconverted coral and CaO/HA residues. Therefore, the experimental evidence suggests that Sr-CHA, Mg-CHA and Sr-Mg-CHA might have formed a core-shell structure.

#### Analyses of conversion and doping ratios

ICP analyses are shown in Table 2. The (Ca + Sr/Mg)/P ratios (2.29–2.53) of the Sr-CHA, Mg-CHA and Sr-Mg-CHA exceed the standard ratio of 1.67 for HA, indicating the existence of unreacted CaCO_3_. After the hydrothermal reaction, the conversion ratios for the Sr-CHA, Mg-CHA and Sr-Mg-CHA were 66.59%, 69.39% and 73.67%, respectively. The doping ratio of Sr in Sr-CHA was 3.39%, while that of Mg was 2.21%. Following co-doping with Sr and Mg, their doping ratios dropped to 1.09% and 0.50%, respectively. The data show that Sr was more easily doped than Mg, and when co-doped, their doping ratios decreased; nonetheless, the doping ratio of Sr was constantly higher.

**Table 2.** The elemental composition of the coral, Sr-CHA, Mg-CHA and Sr-Mg-CHA from ICP

Samples	Ca (wt.%)	P (wt.%)	Sr (wt.%)	Mg (wt.%)	CR^a^	(Ca + Sr/Mg)/P^b^	Sr/(Ca + Sr/Mg)^c^	Mg/(Ca + Sr/Mg)^d^
Coral	35.58	0.02	0.10	0.10	—	—	—	—
Sr-CHA	30.32	9.60	2.34	0.11	66.59%	2.53	3.39%	—
Mg-CHA	33.61	11.06	0.76	0.43	69.39%	2.39	—	2.21%
Sr-Mg-CHA	32.20	11.16	1.37	0.20	73.67%	2.29	1.09%	0.50%

a Conversion ratio of HA from coral.

b (Ca + Sr/Mg)/P represents the combination of (Ca + Sr)/P, (Ca + Mg)/P, and (Ca + Sr + Mg)/P in the Sr-CHA, Mg-CHA and Sr-Mg-CHA, respectively.

c Sr/(Ca + Sr/Mg) represents the combination of Sr/(Ca + Sr) in the Sr-CHA and Sr/(Ca + Sr + Mg) in Sr-Mg-CHA, which means the doping ratio of Sr in HA converted from coral.

d Mg/(Ca + Sr/Mg) represents the combination of Mg/(Ca + Mg) in the Mg-CHA and Mg/(Ca + Sr + Mg) in the Sr-Mg-CHA, which means the doping ratio of Mg in HA converted from coral.

#### Nitrogen adsorption-desorption isotherms

**Figure 3.**
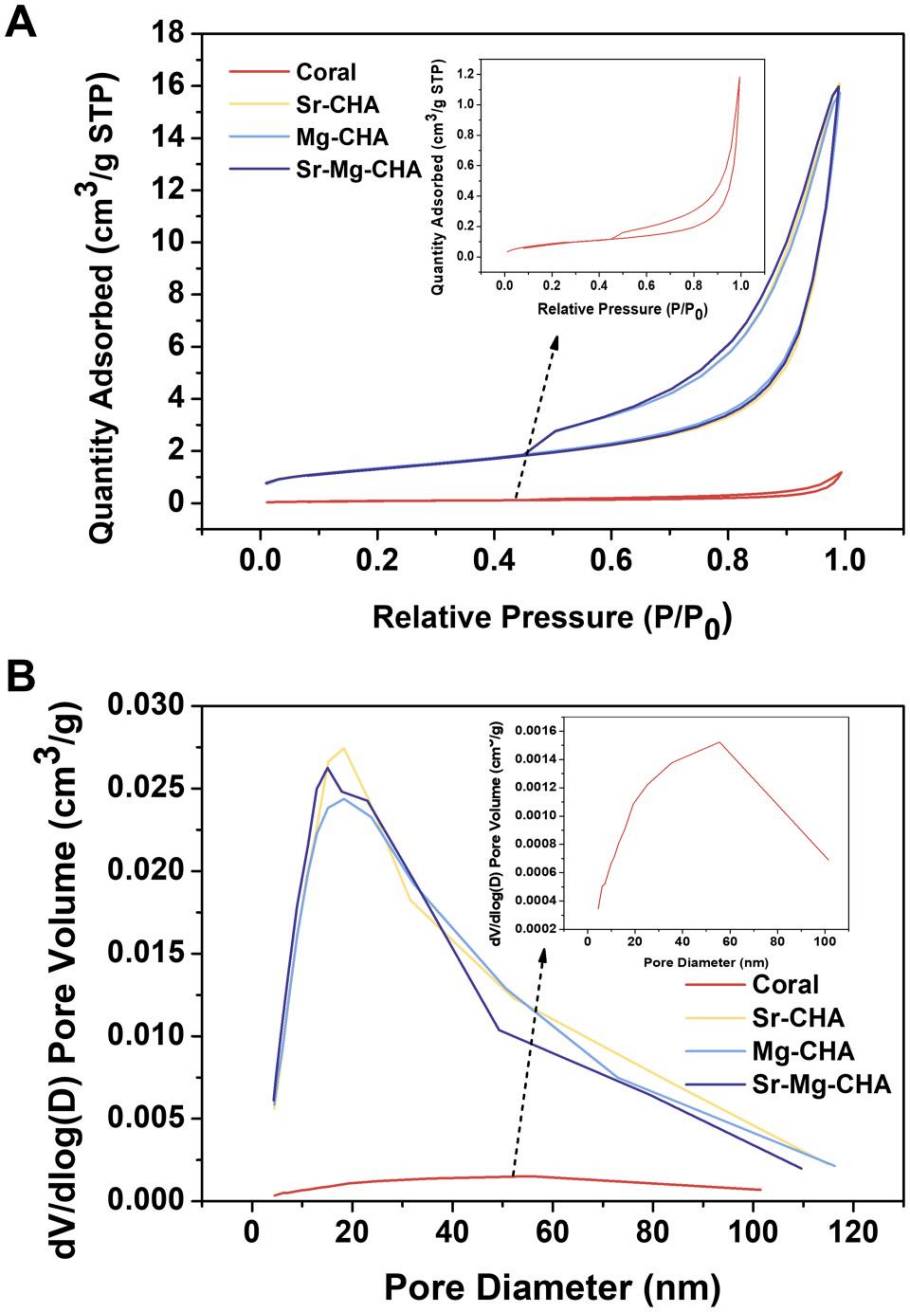
(**A**) Nitrogen adsorption–desorption isotherms and (**B**) pore size distribution curves of the coral, Sr-CHA, Mg-CHA and Sr-Mg-CHA.

The nitrogen adsorption–desorption isotherms (Figure 3A) of the coral, Sr-CHA, Mg-CHA and Sr-Mg-CHA were type IV isotherms according to IUPAC. Figure 3B shows the Barrett-Joyner-Halenda (BJH) adsorption branch analysis of pore size distribution. Pore size distribution curves peak at the average pore size. Table 3 presents the specific surface areas, BJH total pore volumes, and average pore sizes for all samples. The coral, Sr-CHA, Mg-CHA and Sr-Mg-CHA exhibited BET-specific surface areas of 0.34, 4.73, 4.86 and 4.71 m^2^/g, respectively. The pore volumes were 0.0018, 0.0249, 0.0244 and 0.0247 cm^3^/g. The pore diameters were 22.28, 21.86, 20.28, and 21.39 nm. The experimental results demonstrated that, compared to coral, the specific surface area of the Sr-CHA, Mg-CHA and Sr-Mg-CHA increased by nearly 16 times, while the average pore volume rose by around 14 times, and the pore sizes remained essentially unchanged. Mg-CHA displayed the smallest pore volume and highest specific surface area.

**Table 3.** Surface area, pore volume and pore size of the coral, Sr-CHA, Mg-CHA and Sr-Mg-CHA

Samples	Specific surface area (m^2^/g)	BJH Pore volume (cm^3^/g)		Average pore diameter (nm)
Coral	0.34	0.0018	22.28	
Sr-CHA	4.73	0.0249	21.86	
Mg-CHA	4.86	0.0244	20.28	
Sr-Mg-CHA	4.71	0.0247	21.39	

### *In vitro* biocompatibility of the Sr/Mg-CHA

#### Degradation and ion release behavior

**Figure 4.**
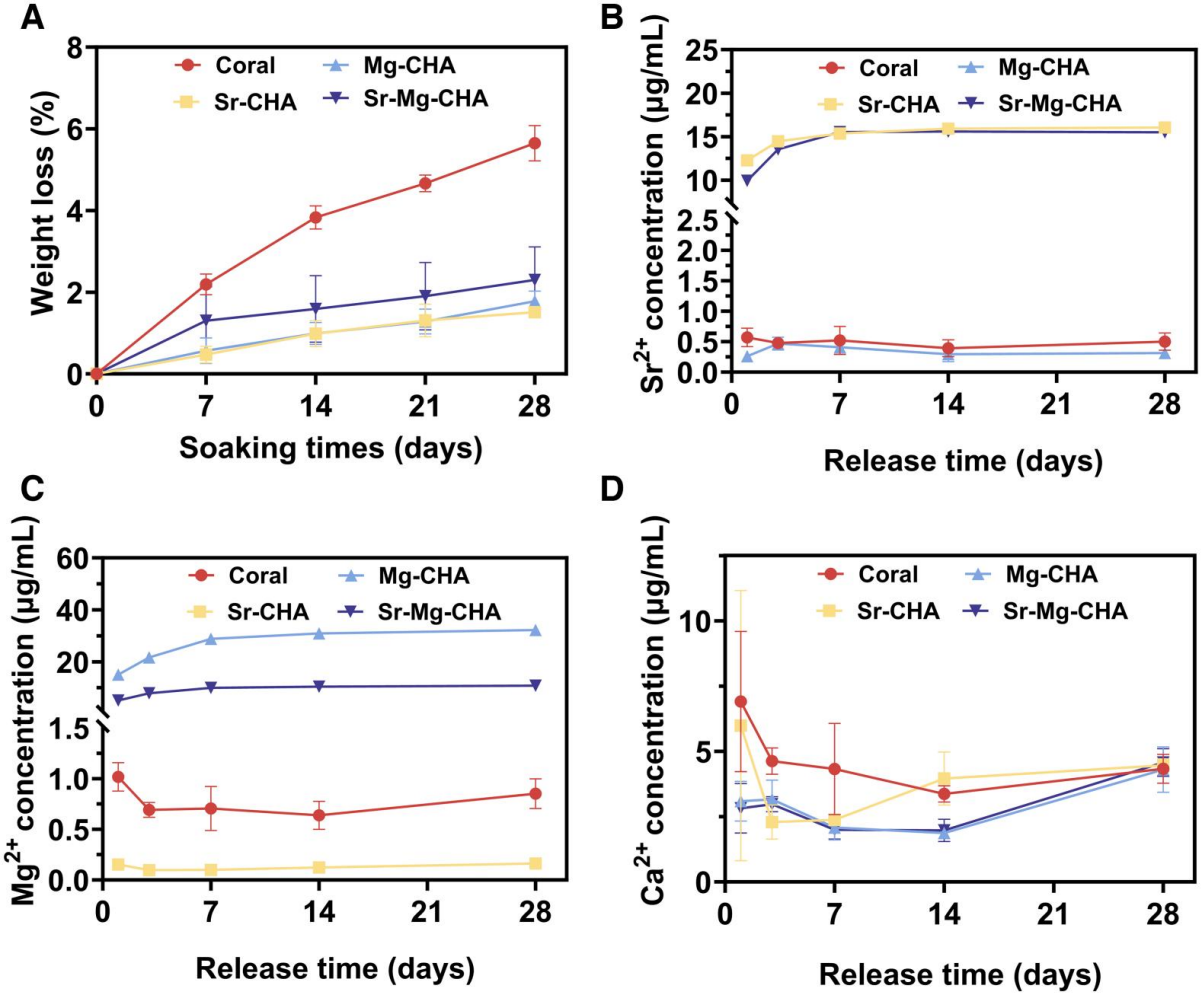
Degradation and ion release behavior of the coral, Sr-CHA, Mg-CHA, and Sr-Mg-CHA (*n* = 3). (**A**) Degradation behavior. (**B**) Sr^2+^ release curves. (**C**) Mg^2+^ release curves. (**D**) Ca^2+^ release curves.

Figure 4 shows samples’ degradation rates in Tris-HCl buffer and ion release behavior for Sr^2+^, Mg^2+^ and Ca^2+^ in PBS. The coral deterioration was maximum after 28 days of immersion, with mass loss near 5.62%. In contrast, the Sr-CHA, Mg-CHA and Sr-Mg-CHA degraded at 2% (Figure 4A). The Sr^2+^ release concentration of the Sr-CHA group exceeded that of the Sr-Mg-CHA in the first 7 days. After 7 days, both groups reached an equilibrium at 15 µg/mL. The coral and Mg-CHA groups showed low Sr^2+^ release levels, staying at 0.4 µg/mL (Figure 4B). The Mg-CHA group released more Mg^2+^ than the Sr-Mg-CHA group in the first week. After 7 days, Mg^2+^ release equilibrium was attained in both groups, with concentrations stabilizing at 30 and 10 µg/mL, respectively, whereas the coral and the Sr-CHA groups maintained values between 0.1 and 1 µg/mL (Figure 4C). Ca^2+^ concentrations remained steady in all groups, averaging around 4 µg/mL throughout 28 days of immersion (Figure 4D). Overall, converting coral into HA will reduce the degradation rate of the material and also decrease the release rate of its Ca^2+^. However, doping with Sr, Mg or Sr-Mg does not significantly affect the rate of Ca^2+^ release from CHA. In addition, the release rate and concentration of Sr^2+^ in the Sr-Mg-CHA and Sr-CHA did not show significant changes after 7 days. While the release rate of Mg^2+^ in Sr-Mg-CHA is remarkably lower than that in Mg-CHA, indicating that the incorporation of Sr^2+^ into Sr-Mg-CHA may have a competitive inhibitory effect on the release of Mg^2+^.

#### Hemolysis rates

**Figure 5.**
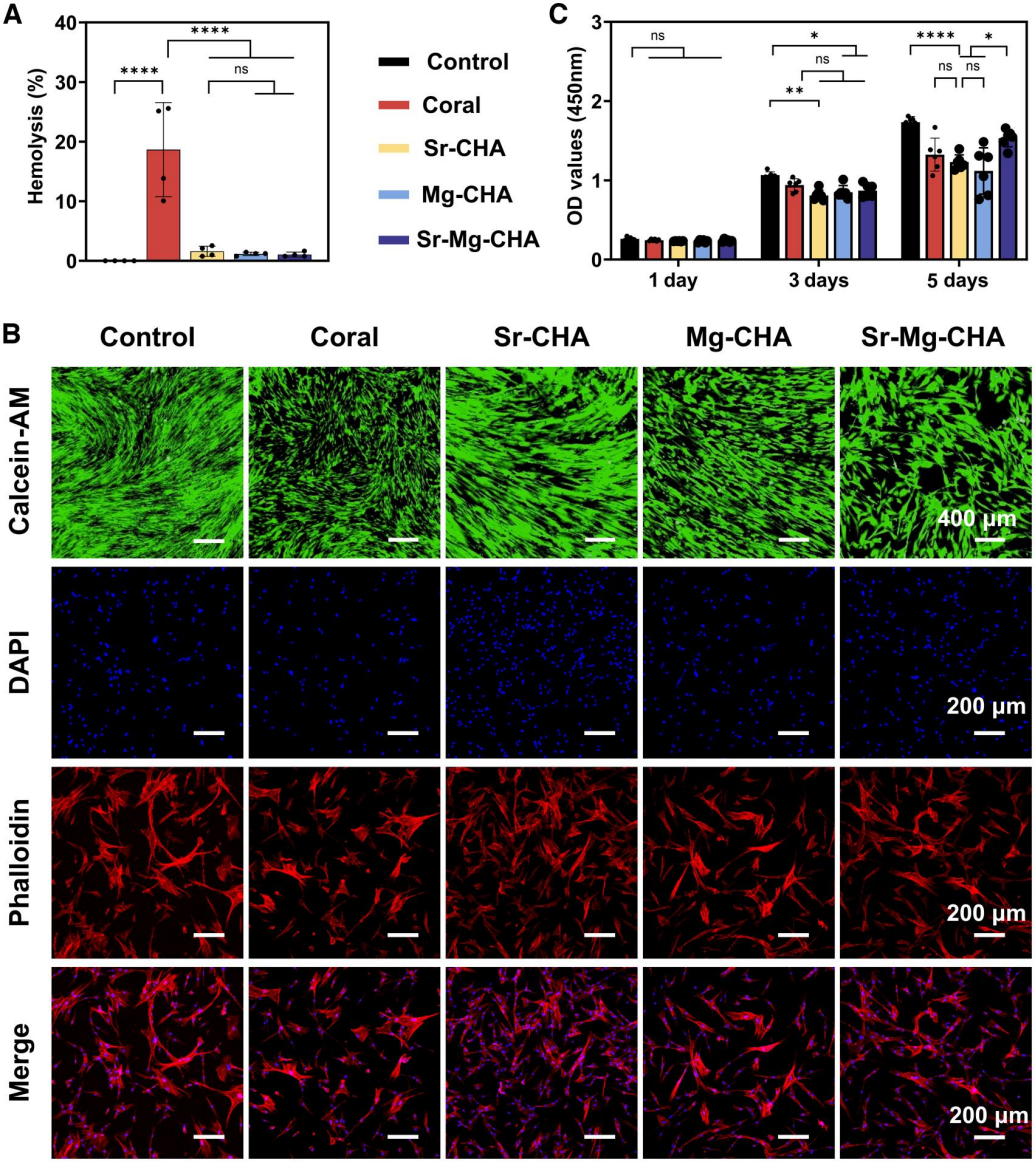
*In vitro* biocompatibility. (**A**) Hemolysis rates (*n* = 4). (**B**) Calcein-AM and cytoskeletal staining of hBMSCs cultivated 2 days in the coral, Sr-CHA, Mg-CHA and Sr-Mg-CHA extracts (*n* = 6). (**C**) hBMSCs activity after co-culturing for 1, 3 and 5 days in the coral, Sr-CHA, Mg-CHA and Sr-Mg-CHA extracts (*n* = 4). Live cells were stained with Calcein-AM, actin filaments were stained with phalloidin and cell nuclei with DAPI. Data are given as mean ± standard deviation (*P *> 0.05 (ns), *P < 0.05, **P < 0.01 and ****P < 0.0001).

The hemolysis test is shown in Figure 5A. The coral group exhibited a considerably greater hemolysis rate (18.7% ± 7.9%) compared to other groups (****P < 0.0001). The Sr-CHA, Mg-CHA and Sr-Mg-CHA groups showed decreased hemolysis rates (1.60% ± 0.85%, 1.16% ± 0.28% and 1.06% ± 0.42%, respectively) compared to the established threshold of 5%, meeting hematocompatibility criteria, indicating the improvement of coral biocompatibility after hydrothermal reaction.

#### Adhesion and proliferation of hBMSCs *in vitro*

The adhesion of hBMSCs was confirmed using Calcein-AM and cytoskeletal staining (Figure 5B). The hBMSCs co-cultured in the coral, Sr-CHA, Mg-CHA and Sr-Mg-CHA extracts for 2 days were stained and analyzed. The coral group had weaker green fluorescence than the control group. However, the Sr-CHA, Mg-CHA and Sr-Mg-CHA displayed significantly higher green fluorescence. Cytoskeleton staining demonstrated that coral group displayed shrinkage and inadequate spreading. However, the Sr-CHA, Mg-CHA and Sr-Mg-CHA groups exhibited improved spreading morphology, especially the Sr-CHA group, demonstrating their higher biocompatibility. Cellular activity was measured after co-culturing hBMSCs with the coral, Sr-CHA, Mg-CHA and Sr-Mg-CHA extracts for 1, 3 and 5 days to determine proliferation (Figure 5C). After 1 day, cell activity was similar between groups. Compared to the control group, cellular activity decreased after 3 and 5 days, although all groups showed significant proliferation and biocompatibility.

### *In vitro* osteogenic properties of the Sr/Mg-CHA

#### ALP staining and ARS staining of hBMSCs in vitro

**Figure 6.**
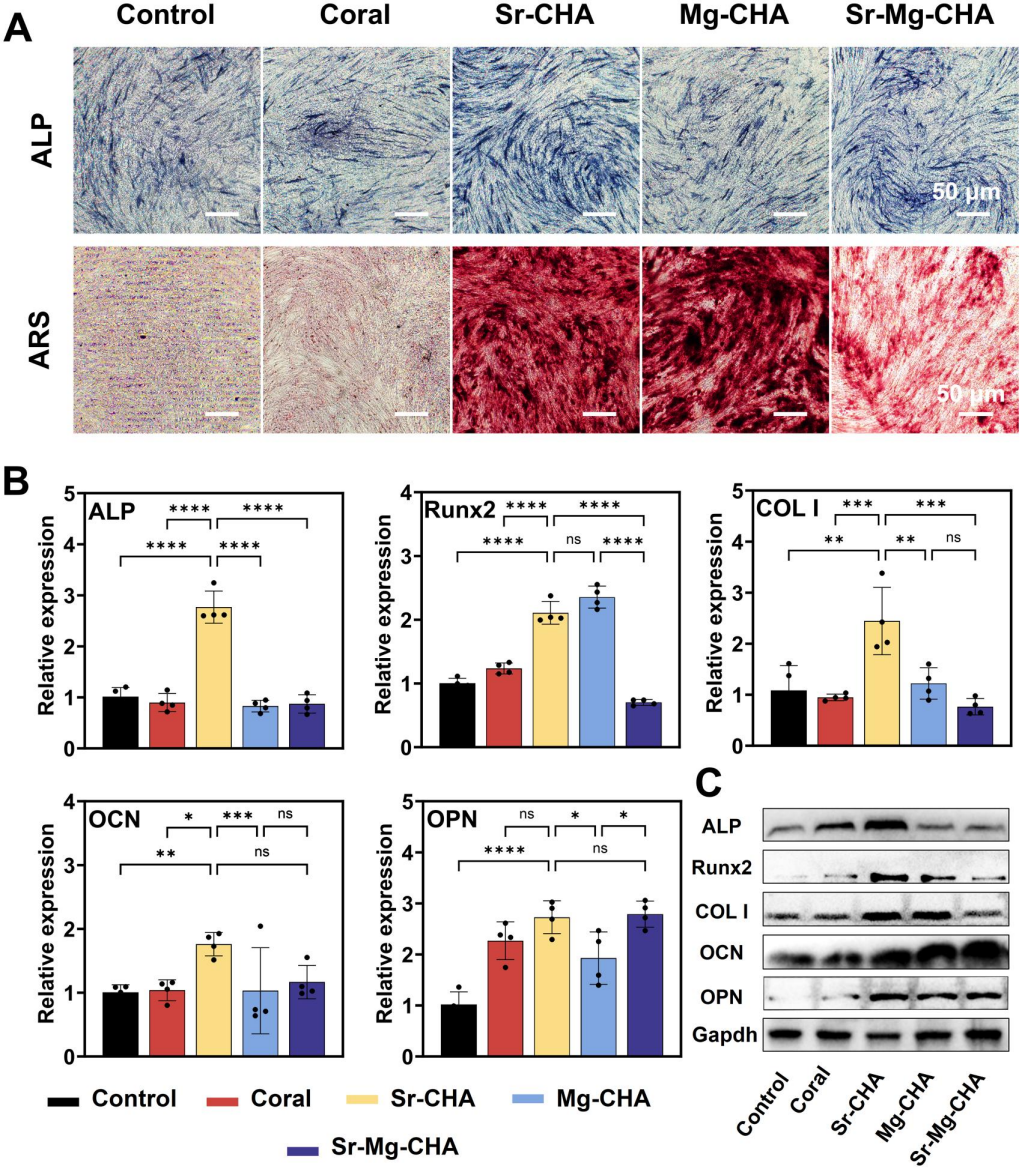
*In vitro* osteogenic differentiation properties (*n* = 4). (**A**) ALP and ARS staining. (**B**) Relative expression of osteogenic differentiation marker genes. (**C**) Protein expression in hBMSCs cultured for 7 days in an osteogenic medium with the coral, Sr-CHA, Mg-CHA and Sr-Mg-CHA extracts. Data are given as mean ± standard deviation (*P *> 0.05 (ns), *P < 0.05, **P < 0.01, ***P < 0.001, and ****P < 0.0001).

Figure 6A shows hBMSCs' ALP activity and ARS staining after 7 days of induced differentiation in an osteogenic medium containing the coral, Sr-CHA, Mg-CHA and Sr-Mg-CHA extracts. The Sr-CHA and Sr-Mg-CHA groups had higher ALP activities. The Sr-CHA showed superior osteogenic differentiation of hBMSCs, while the coral, Mg-CHA and the control inhibited it. The control and coral groups exhibited minimal orange-red calcium nodule formation, according to ARS staining. Simultaneously, Sr-Mg-CHA extracts displayed few nodules. Conversely, the Sr-CHA and Mg-CHA exhibited visible, dense orange-red calcium nodules, especially in the Sr-CHA.

#### Osteogenesis-related genes and proteins of hBMSCs in vitro

Figure 6B shows the expression of osteogenesis-related genes induced by hBMSCs cultured in an osteogenic medium containing the coral, Sr-CHA, Mg-CHA and Sr-Mg-CHA extracts for 7 days. The Sr-CHA group showed significantly higher levels of ALP, COL I and OCN expression compared to other groups (*P < 0.05). The Sr-CHA and Mg-CHA groups secreted the most Runx2 compared to the control, coral and Mg-CHA groups, with no significant differences. Compared to the control group, the expression of OPN was upregulated in the coral, Sr-CHA, Mg-CHA and Sr-Mg-CHA groups, especially in the Sr-CHA and Mg-CHA groups. Western blotting (Figure 6C) showed that hBMSC-induced osteogenesis-related protein expression (e.g. ALP, Runx2, COL I, OCN and OPN) was low in the control and coral groups for 7 days. In contrast, the Sr-CHA, Mg-CHA and Sr-Mg-CHA groups increased osteogenesis-related protein expression in hBMSCs, with Sr-CHA exhibiting the greatest levels. Overall, the Sr-CHA, Mg-CHA and Sr-Mg-CHA groups promoted the expression of genes and proteins and enhanced osteogenic differentiation *in vitro* compared to the coral and control groups. The Sr-CHA group performed best.

### *In vivo* osteogenic properties of the Sr/Mg-CHA

#### Animal modeling and micro-CT analysis

**Figure 7.**
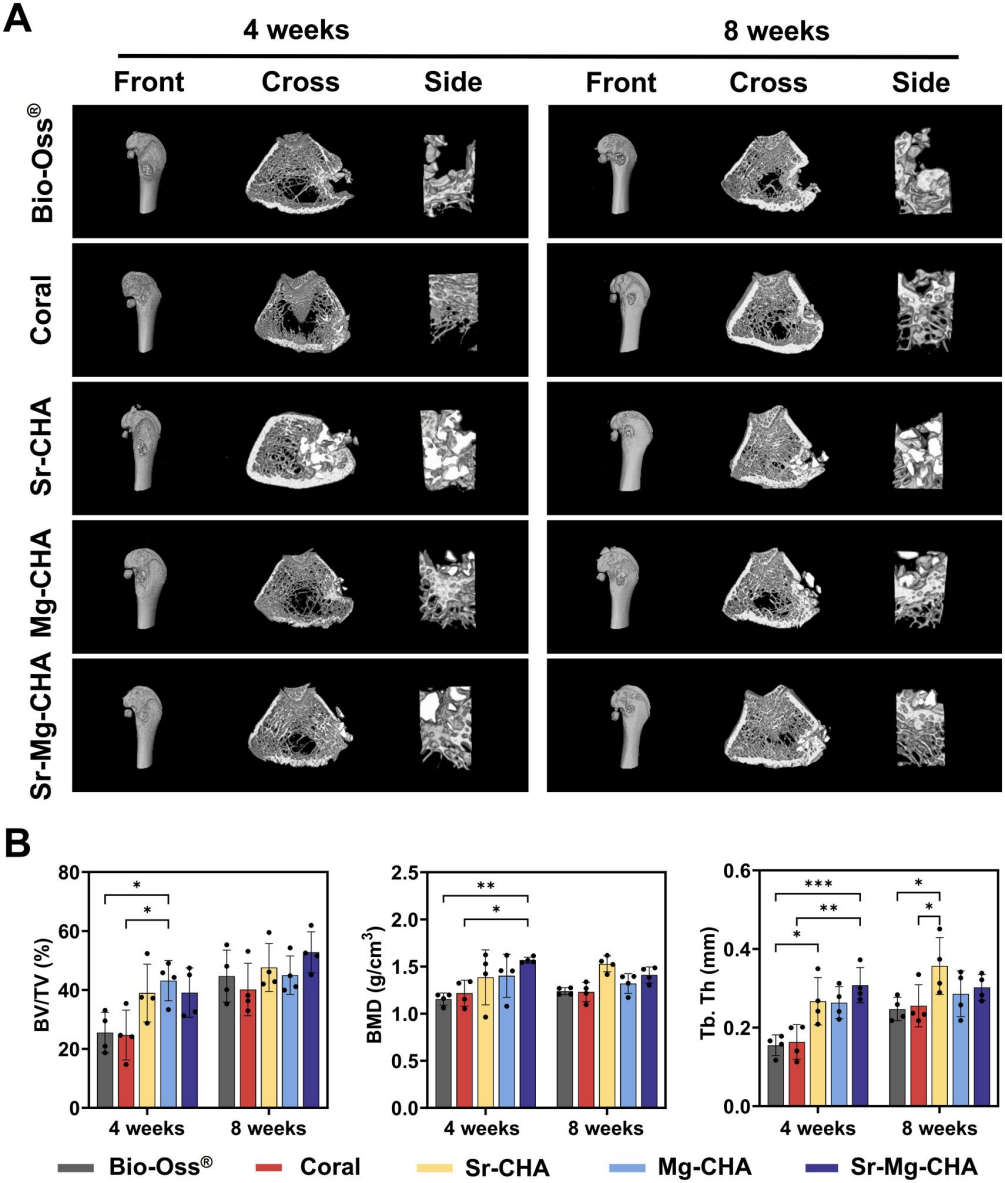
Micro-CT results of the Bio-Oss^®^, coral, Sr-CHA, Mg-CHA and Sr-Mg-CHA implanted into rat femoral condyle defects at 4 and 8 weeks (*n* = 4). (**A**) Reconstructed micro-CT images. (**B**) The quantitative analyses of the osteogenesis indices, including BV/TV, BMD and Tb.Th. Data are given as mean ± standard deviation (*P < 0.05, **P < 0.01, ***P < 0.001).

The rat femoral condyle defects using the Bio-Oss^®^, coral, Sr-CHA, Mg-CHA and Sr-Mg-CHA were reconstructed using 3D Micro-CT (Figure 7A). The reconstructed images at 4 and 8 weeks demonstrate the samples' implantation position, new bone conditions, and *in vivo* degradation. Images show that cancellous bone was not healed 4 weeks after implantation, despite Bio-Oss^®^ and coral basically degradation. The Sr-CHA, Mg-CHA and Sr-Mg-CHA groups had more undegraded material, whereas new bone formation like trabeculae increased, particularly in the cancellous bone. New bone growth was greatest in the Sr-CHA group, which underwent practically complete internal defect repair. All groups showed improved bone tissue regeneration after 8 weeks of implantation. The Sr-CHA, Mg-CHA and Sr-Mg-CHA groups had significant bone tissue regeneration, notably the Sr-CHA group, which repaired almost completely. The Bio-Oss^®^ and coral groups exhibited poor bone tissue regeneration. The reconstructed greyscale maps were analyzed using SkyScan software to quantitatively assess BV/TV, BMD and Tb.Th of the defects (Figure 7B). The Sr-CHA, Mg-CHA and Sr-Mg-CHA groups had significantly higher BV/TV, BMD and Tb.Th than the coral and Bio-Oss^®^ groups after 4 weeks of implantation. At 8 weeks post-implantation, all groups had better bone tissue regeneration. The Sr-CHA, Mg-CHA and Sr-Mg-CHA had higher BV/TV, BMD and Tb.Th than the coral and Bio-Oss^®^ groups, with the Sr-CHA exhibiting the best Tb.Th, supporting the differentiation properties *in vitro* (Figure 6) and reconstructed images (Figure 7A). Moreover, a semi-quantitative analysis of newly formed bone area ratio in Masson-stained histological sections was performed using Image J (Supplementary Figure S3). At 4 weeks, the Sr-CHA, Mg-CHA and Sr-Mg-CHA groups exhibited significant bone formation tendencies (*P < 0.05) compared to the Bio-Oss^®^ and coral groups. By 8 weeks, the Sr-CHA and Mg-CHA groups demonstrated superior osteogenic effects than other groups (**P < 0.01).

#### Histological analysis

**Figure 8.**
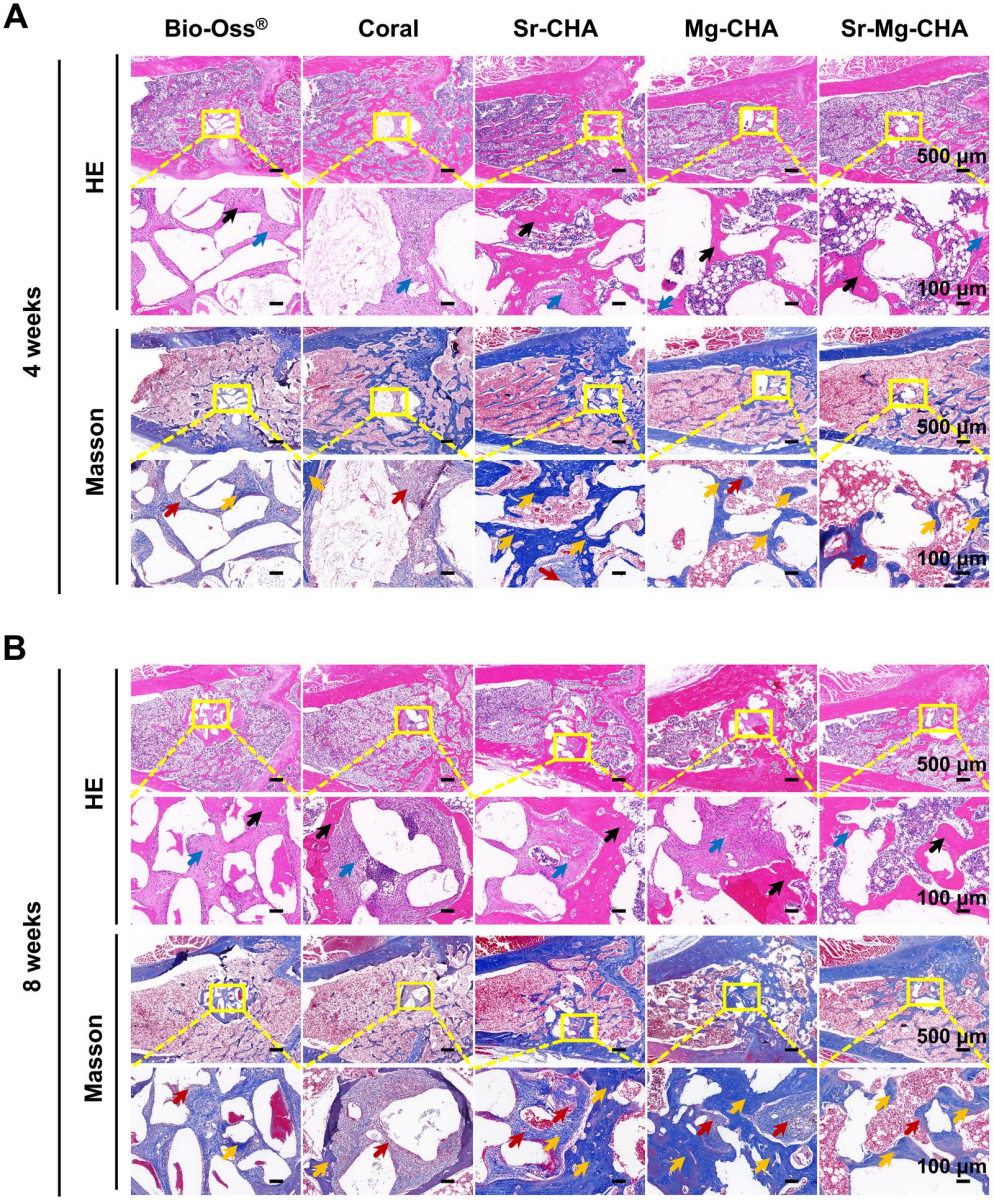
HE and Masson staining of bone tissue with the Bio-Oss^®^, coral, Sr-CHA, Mg-CHA and Sr-Mg-CHA implanted into rat femoral condyle defects (*n* = 4). (**A**) 4 weeks. (**B**) 8 weeks. Woven bone (blue arrows), lamellar bone (black arrows), new collagen (red arrows), mature bone (yellow arrows).

Figure 8 displays HE and Masson's staining results at 4 and 8 weeks after surgery. All groups showed new bone formation after 4 weeks, mostly at the lateral edges and gradually moving toward the defect center. The coral group showed few new bone tissue, collagen fibers and more loose woven bone formation. The Bio-Oss^®^ had some new bone tissue and collagen fibers, and the woven bone was still evident. However, the Sr-CHA and Mg-CHA groups exhibited more newly formed bone tissue and laminar bone structure, especially Sr-CHA. All groups showed the maturation of neonatal bone tissue and mature osteoblasts after 8 weeks of implantation. The Bio-Oss^®^ group had more collagen fibers and better newly formed bone tissue but less mature bone. The Sr-CHA group displayed the highest proportion of newly formed and mature bone tissue, as well as laminar bone, consistent with Micro-CT results (Figure 7).

## Discussion

Coral, a natural bone graft biomaterial, has remarkable biocompatibility and osteoconductivity [16, 18, 20, 30]. However, it faces a degradation mismatch and poor osteoinduction *in vivo* [15, 20]. This study uses a hydrothermal reaction to produce the Sr/Mg-CHA (Figure 1A), a combination of Sr-CHA, Mg-CHA and Sr-Mg-CHA, and assesses its phase composition, functional groups, composition, morphology, degradation and ion release behavior, as well as its bioactivity *in vitro* and bone regeneration *in vivo*. We found that the Sr/Mg-CHA demonstrated better biocompatibility, gene expression, protein expression *in vitro* and osteogenic activity *in vivo* compared to Bio-Oss^**^®^**^ and coral, particularly for the Sr-CHA because of its high specific surface area, pore volume and optimal ion release concentrations.

### The construction of the Sr/Mg-CHA

HA crystal can be formed by a dissolution-reprecipitation mechanism under hydrothermal conditions [35]. HA coordinates Ca^2+^ in two ways. Ca-(PO_4_)_6_ is formed from one Ca^2+^(I) and 6 PO_4_³^−^ tetrahedra. OH^−^ forms an OH-Ca_6_ coordination octahedron with 6 Ca^2+^(II) [36]. HA grows mostly Ca-(PO_4_)_6_ along the *c*-axis and OH-Ca_6_ along the *a* and *b* axes [37]. Smaller cations occupy smaller coordination sites to reduce electrostatic repulsion from nearby anions. Larger cations have more coordination numbers to form ionic bonds with nearby anions [38]. The ionic radius of Ca^2+^, Mg^2+^ and Sr^2+^ are 0.99, 0.72 and 1.13 Å, respectively, which results in Sr^2+^ mainly occupying Ca^2+^(I) coordination sites, whereas Mg^2+^ occupies Ca^2+^(II) of HA during the hydrothermal reaction. Previous studies have also found that the substitution of Ca^2+^(I) with Sr promotes crystal growth along the *c*-axis and inhibits growth along the *a* and *b* axes, resulting in rod-like subgrain elongation and lattice expansion in HA [32, 33]. When Mg substitutes Ca^2+^(II), *a*- and *b*-axis crystal growth is boosted [35, 39, 40]. However, the smaller growth unit OH-Ca_6_ causes lattice shrinkage and shorter Mg-doped nanorods [37]. Meanwhile, Mg adsorption on HA crystals limits *c*-axis growth and makes nanorods shorter and rounder [35, 41]. Overall, it appears that Sr doping in the Sr-CHA promoted crystal growth along the *c*-axis during the hydrothermal reaction, resulting in the longest nanorods (480 ± 85.9 nm). Due to the limited space of OH-Ca_6_ or Mg adsorbed on HA, Mg doping decreased the length (265.5 ± 41.2 nm). Co-doping inhibited Sr-Mg-CHA's Sr and Mg contents, resulting in an intermediate length (394.3 ± 64.8 nm) (Supplementary Figure S2). Mg doping is more likely to generate lattice defects than Sr doping due to the larger ionic radius difference between Ca^2+^ and Mg^2+^ compared to Ca^2+^ and Sr^2+^, leading to irregular nanocluster morphology [35, 42] (Figure 1B).

Pal *et al*. found that mixing with the inner CaCO_3_ layer raised the Ca/P ratio (1.74–1.94) of HA produced on the shell's surface [43]. The Sr/Mg-CHA exhibited Ca/P and (Ca + Sr/Mg)/P molar ratios exceeding 1.67, which can be attributed to 26.5–33.5% unconverted CaCO_3_ in the material (Figure 2 and Tables 1 and 2). This study further found that Sr doping (3.39%) in the Sr-CHA was more facile than Mg doping (2.21%) in the Mg-CHA under the same hydrothermal reaction conditions (Table 2), owing to the smaller ionic radius difference with Ca^2+^ [35, 42, 44]. What is remarkable is that co-doping of Sr and Mg resulted in mutual inhibition (Sr 1.09%, Mg 0.5%). One probable explanation is that when the doping ratio of Mg is <10%, some Mg is doped into HA crystals, resulting in irregular grain formation, while the excess Mg is adsorbed onto the HA surface, blocking Sr doping and reducing the Sr doping ratio [42]. Simultaneously, substituting Ca with Sr, which has a larger ionic radius, causes lattice expansion, which inhibits Mg doping and decreases the doping ratio of Mg [35, 42, 45].

The Sr/Mg-CHA displayed an HA phase, with minor CaCO_3_ peak and no other phases or significant peak shifts (Figure 2A). Prior studies have noted that the peak positions of HA at 28–29°, 32°, and 33° move to the left as the doping ratio of Sr increases to 40–100% [46]. Similarly, if the doping ratio of Sr in HA exceeds 15%, the (002) peak broadens and shifts to lower angular values, with peaks at 2θ ranging from 28° to 29° and 31° to 34° [47]. Additionally, the Ca_9_Sr(PO_4_)_6_(OH)_2_ and Sr_5_(PO_4_)_3_OH phases were identified, with the doping ratio of Sr in HA exceeding 50% [43]. The Ca_7_Mg_2_P_6_O_24_ nanophase peak emerges when the Mg doping ratio in HA reaches 8%. The phase composition of HA stays invariant when the Mg doping ratio is at 5% or below [48]. Consequently, comparison of the findings with those of prior studies confirms that the phase composition of the Sr/Mg-CHA predominantly comprised HA with a minor unconverted CaCO_3,_ as the doping ratios of Sr and Mg (Table 2) were significantly lower than the requirements for phase composition transformations and peak shifts.

The Sr/Mg-CHA exhibited similar functional group characteristics and displayed the CO_3_^2−^ specific functional groups of coral (Figure 2B). The Sr/Mg-CHA spectra showed functional groups of OH^−^ and PO_4_³^−^, which are similar to HA or Sr/Mg-doped HA reported in recent research [22, 31, 45, 48–51], corroborating the XRD patterns (Figure 2A). Previous research indicates that replacing Ca with Mg leads to smaller and denser vacancies in HA, facilitating H_2_O adsorption due to the greater radius difference between Ca^2+^ and Mg^2+^ compared to Sr^2+^ [45, 48]. As expected, the FTIR spectra of the Mg-CHA group were widened and smoothed. For example, a broad absorption band of 2900–3600 cm^−1^ was identified in the Mg-CHA group, which might result from H_2_O absorption in lattice vacancies. Moreover, the minor doping ratio of Mg (0.5%) may explain why Sr-Mg-CHA and Sr-CHA have similar spectral and functional group features (Figure 2A). The change in its functional group depends mostly on Sr.

The surface structural properties of the coral changed significantly after the hydrothermal reaction, although the coral and Sr/Mg-CHA had mesoporous structures (Figure 3A and B). Despite a slight decrease in average pore size, the specific surface area and BJH total pore volume of Sr/Mg-CHA increased 16 and 14 times compared to the coral, respectively (Table 3). These significant increases can be attributed to the crystal growth in the hydrothermal reaction, especially the growth of a large number of nanorods on the coral surface (Figure 1B). Furthermore, the substitution of Ca with Mg in the Mg-CHA, which had a higher proportion of Mg, led to lattice contraction, reduced lattice spacing, smaller grains, and denser voids, resulting in smaller pore size and a slightly increased specific surface area than the Mg-CHA and Sr-Mg-CHA [35].

### Biocompatibility of the Sr/Mg-CHA

Bone tissue regeneration requires biocompatibility for cell proliferation and metabolism. Degradability and ion release behavior affect biomaterial biocompatibility along with chemical composition, phase composition, surface characteristics, etc. [12].

We found that the doping ratio had no effect on the degradation rates of the Sr/Mg-CHA, which was 2%, 1.8 times lower than coral (Figure 4A). However, Sr and Mg doping ratios greatly affected ion release (Figure 4B and C). Sr^2+^ release from the Sr-CHA was higher than the Sr-Mg-CHA within 7 days but stabilized at 15 µg/mL (0.1712 mM) after this period (Figure 4B). Mg-CHA ion release stabilized at 30 µg/mL (1.234 mM) and Sr-Mg-CHA at 10 µg/mL (0.411 mM) after 7 days (Figure 4C), with Mg-CHA releasing more than Sr-Mg-CHA across the 28-day immersion duration. Kruppke *et al*. showed that 0.4–1.8 mM Sr^2+^ concentrations promote hBMSCs proliferation and differentiation [52]. Moreover, Li *et al*. found that Mg^2+^ at concentrations of 2–10 mmol/L was not cytotoxic to hBMSCs, whereas concentrations of 4–6 mmol/L significantly promoted their proliferation and osteogenic differentiation [53]. As a result, the degradation and ion release concentration of the Sr/Mg-CHA met biocompatibility criteria, making it biocompatible (Figure 5). Despite numerous studies showing that coral is biocompatible [16, 17, 54]. Unexpectedly, coral had a higher hemolysis rate (18.7% ± 7.9%, ≥5%) than the ISO 10993 standard (Figure 5A), despite exhibiting good cell proliferation and adhesion. One reasonable explanation is that at physiological pH (7.4), negatively charged erythrocyte membranes (isoelectric point 3.5) may interact with positively charged coral surfaces (isoelectric points 8–10), causing membrane instability and rupture and increasing hemolysis risk. The negatively charged Sr/Mg-CHA surface (isoelectric point 4.8), similar to HA, repels the erythrocyte membrane, preventing direct contact and hemolysis [55–57]. In contrast, hBMSCs are nucleated cells with complex cell membranes and increased sensitivity to external stimuli. Consequently, corals exhibited hemolytic qualities, while they also possessed substantial adhesion and proliferation on hBMSCs (Figure 5B and C). It is acknowledged that while Sr/Mg-CHA's degradation has benefits, rapid dissolution might weaken the Sr-Mg-CHA’s structure before full bone regeneration, and inconsistent ion release could affect biological responses.

### The mechanism of the Sr/Mg-CHA for enhanced osteogenesis and bone regeneration

Prior research shows that Sr^2+^ stimulates hBMSCs differentiation via the OPG/RANKL/RANK and NF-κB signaling pathways, inhibits osteoclast activity and enhances ALP activity and osteogenesis-related gene expression, such as Runx2, COL I, OPN and ALP [27]. Mg^2+^ enhances hBMSCs proliferation, adhesion and osteogenic differentiation by stimulating the Wnt/β-catenin signaling pathway [28]. Kruppke *et al*. found that 0.4–1.8 mM Sr^2+^ promoted the proliferation and differentiation of hBMSCs [52]. Braux *et al*. also found that osteoblast marker expression peaked at doses of roughly 0.05–1 mM [58]. Ran *et al*. indicated that the doping ratio of Sr between 3% and 7% in HA can enhance the activity and differentiation of osteoblasts [27]. Another study found that Mg^2+^ increased the expression of osteogenesis-related genes Runx2, ALP, OPN and OCN in hBMSCs at concentrations from 100 to 500 mg/L (4.11–20.55 mM) compared to the control group, with the highest effect at 4.11 mM [59].

Proteins are essential molecules for cellular recognition and adhesion, delivering critical signals for cell attachment, proliferation and differentiation [12, 60]. The BSA adsorption increases with the specific surface area and pore volume of n-HA [61]. Mg-HAp coatings doped with Mg^2+^ on titanium alloy substrates have an increased specific surface area, enhancing protein absorption and promoting bone formation [62].

As shown in Table 3, the hydrothermal conversion process increases the specific surface area and pore volume of doped CHA nearly 16- and 14-fold, respectively, compared to the coral. It is presumed that the Sr/Mg-CHA's higher specific surface area and pore volume can increase serum protein adsorption, such as BSA, adhesion-associated integrin ligands, facilitate cellular adhesion and proliferation and enhance osteogenesis by adsorbing more osteogenic growth-associated factors, such as BMP-2, which was essential for osteoblast development and differentiation, boosting osteogenic effects *in vitro* and *in vivo* [63, 64]. In addition, Sr/Mg-CHA had more compatible degradation qualities during osteogenesis than coral and Bio-Oss^®^, which degraded too quickly and interfered with bone development. These features supported bone tissue proliferation and host bone tissue integration (Figures 7 and 8). The Sr^2+^ release equilibrium concentration in the Sr-CHA and Sr-Mg-CHA groups remained at 15 μg/mL (0.1712 mM), a concentration close to 0.4–1.8 mM, which was beneficial to osteogenic differentiation. The Sr-CHA showed a higher Sr^2+^ concentration than Sr-Mg-CHA during the first 7 days. The Mg-CHA and Sr-Mg-CHA groups produced Mg^2+^ at equilibrium concentrations of 30 μg/mL (1.234 mM) and 10 μg/mL (0.411 mM), respectively, which were below the effective range for osteogenic differentiation (Figure 4B and C). Unconverted CaCO_3_ in Sr/Mg-CHA serves as a sustained-release reservoir, hastening late-stage degradation, shortening the cycle, and boosting bioactivity by regulating Ca^2+^ release. Its degradation and ion-release patterns align with osteoblast behavior, promoting cell proliferation and differentiation. Additionally, nanostructural variations, like nanorod dimensions and shapes in Sr/Mg-CHA, can influence extracellular matrix mineralization and cell behavior, with specific nanostructures guiding cell orientation and function. Due to these synergistic effects, the Sr/Mg-CHA group surpasses coral and Bio-Oss^®^ in ARS and ALP staining intensity, osteogenesis-related gene and protein expression, *in vitro* osteogenic differentiation, and *in vivo* bone formation (Figures 5–8).

### The characteristics and limitations of this study

HA is biocompatible and osteointegrated but nondegradable, which may hinder bone remodeling. Sr/Mg-doped HA, though more soluble, has limited degradation and clinical use. *In vivo*, Mg-doped HA (Mg-HA) showed granulomas after 45 days in a rat model, possibly due to insufficient degradation, despite better bone mineralization [65]. Thus, Sr/Mg-doped HA is rarely used clinically. Although Liu *et al*. initially prepared Sr-doped coralline HA, it still exhibits insufficient *in vivo* degradation and lacks systematic osteogenic studies [14]. Additionally, there is no research on coral with Mg doping or Sr-Mg co-doping.

Consequently, a Sr/Mg-CHA was designed. The material possesses a unique porous “core-shell” structure that is formed by hydrothermal conversion of CaCO_3_ from natural coral. The external Sr/Mg-CHA shell (66.5–73.5% conversion) provides active sites for bone integration, promoting osteoblast adhesion and differentiation. The inner unconverted CaCO_3_ (26.5–33.5%) rapidly dissolves in the body's mildly acidic environment, with a rate of up to 40–50% resorption of their volume after a year or more over a period [66]. The inherent porosity of the coral (100–500 µm) serves to accelerate the overall degradation of the material. Unlike previous research, this study combines partially converted HA with coral ion doping, considering natural porosity and controllable degradability. Using a two-step ion-exchange method after partial HA conversion, Sr/Mg-CHA retained coral's porous nature and achieved controllable degradability. The study also systematically explained the synergistic mechanism of degradation and osteogenesis, addressing a key gap in coral-based biomaterials research.

However, our study does have certain limitations. For one thing, there is insufficient molecular-level evidence to confirm the effects of Sr^2+^ and Mg^2+^, such as data from protein expression analysis or signaling pathway studies, which might affect the credibility of our conclusions. To address this, we plan to use methods like western blotting and ELISA to analyze related proteins of the signaling pathway or employ fluorescent reporters to monitor pathway activation, thereby gaining a deeper understanding of the mechanisms of Sr^2+^ and Mg^2+^.

Another thing is the lack of in-depth research on the long-term degradation performance of the Sr/Mg-CHA. Previous studies have revealed that Sr substitution for Ca in HA caused structural disorder, reducing crystallinity and creating defect. The dissolution rate rises with Sr content, starting mainly at defects and Sr-rich grain boundaries [67, 68]. These findings inform Sr/Mg-doped CHA's long-term degradation and guide material design by linking microstructure to degradation. We will conduct long-term *in vitro* tests, measuring Sr^2+^/Mg^2+^ release with ICP-MS/ion chromatography. Mechanical properties will be compared to native bone and commercial HA, while clinical relevance will be validated in large-animal models. Fluorescent labeling will track mineralization and assess biocompatibility. These steps will improve understanding and support Sr/Mg-CHA's application in bone tissue engineering.

## Conclusion

This study successfully synthesized CHA doped with Sr, Mg, and a combination of both by hydrothermal reaction. The Sr-CHA, Mg-CHA and Sr-Mg-CHA were biocompatible, promoting hBMSCs proliferation and adherence and upregulating osteogenic-related gene and protein expression (e.g. ALP, Runx2, COL I, OCN and OPN) than the coral *in vitro*. The rat femoral condylar defect healing model confirmed that the osteogenic properties of these doped materials promote bone defect repair compared to coral and Bio-Oss^®^  *in vivo*. These doped materials have a large specific surface area and pore volume, especially the Sr-CHA, which has excellent osteogenesis *in vitro* and *in vivo* due to its appropriate Sr^2+^ release concentration for osteogenic differentiation, providing new perspectives in the field of bone regeneration.

## Supplementary Material

rbaf036_Supplementary_Data
