# Supplementary material for: Strontium/magnesium-doped coralline hydroxyapatite for bone regeneration
Source: Regen Biomater. 2025 May 21;12:rbaf036. doi: 10.1093/rb/rbaf036 (PMC12362243; doi:10.1093/rb/rbaf036)
Supplement: rbaf036_Supplementary_Data [file rbaf036_supplementary_data.zip › Final Supporting Information (RB-2025-067.R1)-clean version.docx]

**Supporting Information**

**Strontium/Magnesium-Doped Coralline Hydroxyapatite for Bone Regeneration**

Bixiu Chen^a, 1^, Liyan Zhang^a, 1^, Zhou Zhong^b, 1^, Chunyu Liu^a*^, Haobo Pan^a, b*^

**^a^** Shenzhen Key Laboratory of Marine Biomedical Materials, CAS-HK Joint Lab of Biomaterials, Shenzhen Institute of Advanced Technology, Chinese Academy of Sciences, Shenzhen, 518055, PR China

^b^ Shenzhen Healthemes Biotechnology Co., Ltd, Shenzhen, 518102, P.R. China

^1^ Authors contributing equally to this article and joint first authors

^*^ Corresponding author:

Prof. Haobo Pan, E-mail: [hb.pan@](mailto:qiaoweihong@dlut.edu.cn)siat.ac.cn

Assoc. Prof. Chunyu Liu, E-mail:cy.liu@siat.ac.cn

**Section S1: Introduction**

1. **Comparative Analysis of HA-Based Biomaterials: Composition, Properties, and Challenges**

**Table S1.** Comparative Analysis of HA-Based Biomaterials: Synthesis Methods, Composition, Properties, In Vitro, In Vivo, and Challenges

| **Materials** | **Synthesis Methods** | **Composition** | **Properties** | **In Vitro** | **In Vivo** | **Challenge** |
| --- | --- | --- | --- | --- | --- | --- |
| Sr-doped HA/CS composite  Coatings [1] | —Chemical precipitation method,  —Spin-coating technology | Sr-doped HA and  Chitosan | —Dense and uniform, coating,  —Adhesion Strength : from 12.41 MPa (pure HA/CS) to 13.33 MPa (4% Sr-doped HA/CS) | —BMSCs,  —Good compatibility,  —ALP activity | NA | —Insufficient uniformity and unstability,  —Lack of reliability and reproducibility of in vitro and in vivo experimental results |
| Sr-doped Hydroxyapatite  Porous Scaffold [2] | —3D printing, —Sintering | Sr-doped HA | —Interconnected pores (300–400 µm),  —Compressive Strength: increased by 30% compared to pure HA scaffolds (4-6 MPa),  —Degradation rate: 20-27% | —rBMSCs,  —Good compatibility,  —ALP staining | NA | —Lack of in vitro ion release studies,  —Lack of adequate in vitro biological performance and potential in vivo biological performance studies |
| Sr-substituted hydroxyapatite (SrHA) powder [3] | —Classical neutralization method,  —Sieving,  —Cellulosic sponges | Sr-doped HA | —4.52 ± 1.40 MPa for SrHA scaffolds,  —45 % porosity,  —Specific Surface Area: 80 m²/g | —Higher solubility compared to HA,  —Sr^2+^ release are constant after 1 week (Hanks’ balanced salt solution) | NA | —Lack of the biological performance in vitro and potentially in vivo |
| Strontium-doped hydroxyapatite polysaccharide [4] | —Wet chemical precipitation,  —Blend,  —Trisodium trimetaphosphate cross-linking | Sr-doped HA and polysaccharide | —No significant change in crystallinity for 8% Sr substitution, but broadening of peaks for 50% Sr substitution | —MSCs,  —Good compatibility,  —Upregulated OPN at Day 3, but not at Day 7 | —12-week-old Balb/c mice,  —2 and 4 weeks,  —Bone formation，  —Blood vessel formation | —Not long-time animal evaluation,  —Not degradation, proliferation, and protein expression studies in vitro |
| Magnesium incorporated hydroxyapatite  [5] | —Wet chemical Precipitation | Mg-doped HA | —Increased in the a lattice,  —Decrease in the c lattice,  —Crystallinity decreased: smaller in Mg-doped HA compared to pure HA | NA | NA | —Lack of in vitro ions release studies,  —Not in vitro and in vivo biological performance studies |
| Magnesium incorporated hydroxyapatite  [6] | —Chemical co-precipitation method | Mg-HA | —Hydrodynamic diameter: 158.3 ± 18 nm  —Potential: −17.77 ± 3.4 mV,  —Crystal Structure: an average grain size of about 93.3 nm | —Antimicrobial Activity: gram-positive, gram-negative, fungal strains | NA | —Not mentioned cytotoxicity,  —Not in vitro and in vivo biological performance studies |
| Magnesium-substituted hydroxyapatite  [7] | —Wet precipitation method  —Extruded microspheres method | Mg-HA | —HA: 3.445 m²/g, MgHA: 2.283 m²/g, | NA | —At 45 days, mild chronic granulomatous inflammation，  —did not enhance bone regeneration compared to HA | —not sufficiently properties characterization,  —no analysis of degradation and ion release,  no in vitro experiments |
| Sr,Mg co-doping of calcium hydroxyapatite  [8] | —Hydrothermal synthesis  —Sintering | SrMg-HAP | —α-Mg and HA phases,  —increased yield tensile strength,  —Corrosion rate increased with increasing HA content | NA | NA | —Lack of in vitro ions release studies,  —Not in vitro and in vivo biological performance studies |

**Section S2: Extended Experimental Protocols**

**S2.1 In vitro cellular response of hBMSCs to Sr/Mg-CHA**

**S2.1.1 Adhesion and proliferation**

hBMSCs were cultured in α-MEM medium with 10% FBS at a density of 5×10^5^ cells per well in 12-well plates. After 1 day, the cell medium was substituted with the extracts of the coral, Sr-CHA, Mg-CHA, and Sr-Mg-CHA groups, along with the control. After 2 days, the cells were washed twice with phosphate-buffered saline (PBS, PB180327, Hyclone, China) and co-cultured with Calcein AM (C2012-0.5 mL, Beyotime, China) for 1 h. The cells were then evaluated for cell activity using an Orthogonal Fluorescence Microscope (CKX53SF, Olympus, Japan).

hBMSCs were cultured in α-MEM medium with 10% FBS at a density of 1.5×10^4^ cells per well in 24-well plates. After 1 day, the cell medium was substituted with the extracts of the coral, Sr-CHA, Mg-CHA, and Sr-Mg-CHA groups, along with the control. After 2 days, the cells were rinsed twice with PBS, treated with 4% paraformaldehyde (BL539A, Biosharp, China) for 30 min, and incubated in Triton X-100 (P0096-100 mL, Beyotime, China) for 10 min at room temperature. The cells were subsequently washed twice with PBS, after which phalloidin (Actin-Tracker Red-555, C2203S, Beyotime, China) was added and incubated overnight at 4°C to stain the cytoskeleton. After 4 washes with PBS, the nuclei of cells were stained with DAPI (C1002, Beyotime, China) for 10 min at room temperature, then washed 3 times with PBS and photographed with a Confocal Laser Scanning Microscope (CLSM, A1/C2-DUVB, Nikon Instruments, Japan) to assess cell spreading morphology.

The proliferation of hBMSCs was evaluated using a Cell Counting Kit-8 (CCK-8, M0856-100, MIKX, China) by measuring the cell concentration. The hBMSCs were cultured in α-MEM medium with 10% FBS at a density of 1×10^4^ per well in 96-well plates. After 1 day, the cell medium was substituted with the extracts of the coral, Sr-CHA, Mg-CHA, and Sr-Mg-CHA groups, along with the control, and it was changed every 2 days. The medium was substituted with serum-free 10% CCK-8 and incubated for 2 h in a light-free incubator for 1, 3, and 5 days. A Multiskan Spectrum Microplate Spectrophotometer (Bio-Rad 680, Berkeley, USA) measured absorbance at 450 nm.

**S2.1.2 ALP and ARS staining**

The hBMSCs were grown at a density of 5 × 10^8^ per well in 6-well tissue culture plates supplemented with α-MEM medium with 10% FBS in culture incubator. After 80% fusion, α-MEM medium was replaced with the conditioned osteogenic induction medium formulated with coral, Sr-CHA, Mg-CHA, and Sr-Mg-CHA extracts mixed with 1.129% osteogenic induction medium (OIM), along with the control to induce hBMSC differentiation for 7 days. After 7 days, the conditioned osteogenic induction medium was removed, and the cells were washed twice with PBS and then fixed with 4% paraformaldehyde for 30 min. Afterward, the BCIP/NBT Alkaline Phosphatase Chromatography Kit (C3206, Beyotime, China) and Osteogenic Assay Kit (SZ2053, MIKX, China) were then used for alkaline phosphatase staining (ALP) and alizarin red staining (ARS). ALP measures early osteogenic differentiation marker expression, while ARS measuresZAF deposition. Fluorescence microscopy was used to examine the differentiation of hBMSCs.

**S2.1.3 Expression of the osteogenesis-related genes**

The expression of osteogenesis-related genes of hBMSCs in the conditioned osteogenic induction medium was assessed by quantitative reverse transcription polymerase chain reaction (qRT-PCR). The HiPure Total RNA Kit (R4114-03, Magen, USA) was used to extract total RNA from hBMSCs after 7 days of osteogenic stimulation by section S2.1.2. RNA was reverse transcribed into cDNA using the ARevert Aid First Strand cDNA Synthesis Kit (MKG840, MIKX, China). PCR ampliffcation reactions were performed on the cDNA using 2× Polarsignal qPCR mix (MKG800-10, MIKX, CHINA) to obtain the cycle number CT values. The relative mRNA expression was normalized based on 2^–ΔΔCt^ (ΔCt = Ct (target gene) – Ct (reference gene), ΔΔCt = ΔCt (experimental group) – ΔCt (control group)). GAPDH was use as a reference gene [9]. The primer sequences employed are summarized in **Table S2** [9, 10].

**Table S2.** Primers for RT-qPCR analysis. (F: forward primer, R: reverse primer).

| Genes | Primer | Primer sequences |
| --- | --- | --- |
| GAPDH | F | TTCGACAGTCAGCCGCATCTT |
|  | R | ATCCGTTGACTCCGACCTTCA |
| ALP | F | ACCACCACGAGAGTGAACCA |
|  | R | CGTTGTCTGAGTACCAGTCCC |
| Runx2 | F | TGGTTACTGTCATGGCGGGTA |
|  | R | TCTCAGATCGTTGAACCTTGCTA |
| OCN | F | CGCTACCTGTATCAATGGCTGG |
|  | R | CTCCTGAAAGCCGATGTGGTCA |
| OPN | F | CGAGGTGATAGTGTGGTTTATGG |
|  | R | GCACCATTCAACTCCTCGCTTTC |
| COL I | F | GAGGGCCAAGACGAAGACATC |
|  | R | CAGATCACGTCATCGCACAAC |

**2.2.4 Western blotting**

Western blot (WB) was used to assess osteogenesis-related protein expression of hBMSCs in the conditioned osteogenic induction medium after 7 days, according to section S2.1.2. Briefly, hBMSCs were collected and centrifuged for 10 min at 1500 rpm. After washing with PBS twice, the cells were lysed in 200 μL of RIPA buffer (DB258, MIKX, China) with 1% phosphatase (DB615, MIKX, China), protease (DB612A, MIKX, China), and PMSF (DB244, MIKX, China) inhibitors on ice and vortexed for 30 min. The supernatants were collected, and the protein concentration was determined using the BCA Protein Assay Kit (P0009, Beyotime, China), then added to 5% SDS-PAGE protein loading buffer 5X (P0285-15m, Beyotime, China), boiled for 10 min, and stored at -80°C. The protein samples were subjected to sodium dodecyl sulfate-polyacrylamide gel electrophoresis (SDS-PAGE) and transferred to PVDF membranes (Merck Millipore, USA). The membranes were blocked with 5% BSA (Sigma-Aldrich, USA) and incubated overnight at 4°C with primary antibodies diluted in PBS. Primary antibodies used included rabbit anti-GAPDH (128915, Abcam, USA), rabbit anti-Runx2 (23981, Abcam, USA), rabbit anti-ALP (381009, Zenbio, China), rabbit anti-COLI (138492, Abcam, USA), rabbit anti-OPN (63856, Abcam, USA), and rabbit anti-OCN (614487, Zenbio, USA). After three washes with 1× TBST, the membranes were incubated with HRP-conjugated secondary antibody (M21002L, Abmart, China). The blots were then enhanced with chemiluminescence (ECL) substrates (MK-S400, MIKX, China) and imaged using the Biomolecular Imager 680 (Typhoon 5, GE Healthcare, USA).

**S2.2 The procedure of hemolysis for the Sr/Mg-CHA**

A diluted blood sample was made by mixing 8 mL of anticoagulated blood with 10 mL of saline. Positive controls were made by adding 10 mL of pure water to each tube, whereas negative controls were made with saline. Three tubes and a 10 mL saline control tube were prepared simultaneously. After 30 min incubation at 37°C on a shaker (THZ-103B, Shanghai Yiheng, China), all tubes except the blank tube were augmented with 0.2 mL of diluted blood per 10 mL of saline. Then each tube's contents were gently mixed. The tubes were then placed in a constant temperature shaker at 37°C for 1 h and gently inverted twice at 30-min intervals to ensure complete contact between the blood and the substance. After incubation, the tubes were gently stirred, and the solution was transferred to a new centrifuge tube for 5 min at 800 g. After transferring the supernatant to a 96-well plate, a spectrophotometer (Bio-Rad 680, Berkeley, USA) assessed absorbance at 545 nm to assess hemolysis.

**S2.3 Histological Staining Procedures**

Following the administration of the implant material (4w and 8w), the rats were euthanized using excessive volatile anesthetic (H19980141, isoflurane, supplied by Tianjin Ruipu Biotechnology Co., LTD). The femur was then removed and fixed in 10% formalin solution for a period of 12 h. Thereafter, the femur was decalcified using a decalcifying solution (BKMAMLAB, 110702062, China) for a duration of one month. The samples were then subjected to a process of gradient dehydration, employing 75%, 80%, and 95% anhydrous ethanol for a duration of 60 min. This was followed by soaking in transparent xylene (10023418, Sinopharm Group Chemical Reagents Co., LTD., China) on two separate occasions, each lasting 40 min. The organ tissue was then embedded in paraffin wax to create a wax block that was embedded in tissue.

**S2.3.1 HE dyeing**

Paraffin sections were successively placed into xylene I (20 min), xylene II (20 min), anhydrous ethanol I (5 min), anhydrous ethanol II (5 min), and 75% alcohol (5 min) in a gradient dewaxing process. The sections were then washed after the dyeing process. The slices were then immersed in hematoxylin (HE dyeing solution, BH0001, Wuhan Bolf Biotechnology Co., LTD., China) for a period of 3**–**5 min. Thereafter, the excess dye solution was rinsed with tap water. The slices were then differentiated using 1% hydrochloric acid (10011008, Sinopharm Group Chemical Reagent Co., LTD, China) for several seconds, after which they were rinsed with tap water. The next step is to use 0.6**–**0.7% ammonia aqueous solution (10002118, Sinopharm Group Chemical Reagent Co., LTD.) to return the blue color, after which the slices should be rinsed with running water for a few seconds. Paraffin sections were then dehydrated with gradient alcohol (85% ethanol, 95% ethanol) and dyed in eosin solution for 5 min. The paraffin sections were then added to anhydrous ethanol I for 5 min, anhydrous ethanol II for 5 min, anhydrous ethanol III for 5 min, and n-butanol (10005218, Sinopsin Group Chemical Reagents Co., LTD., China) for 5 min, xylene I for 5 min, xylene II for 5 min to make them transparent, then remove the sections from the xylene to dry slightly. Neutral gum (B0044, Wuhan Bolf Biotechnology Co., LTD., China) seal. The nucleus is blue and the cytoplasm is red.

**S2.3.2 Masson dyeing**

Paraffin sections were dewaxed according to the HE staining method outlined above. Thereafter, the paraffin sections were immersed in a potassium dichromate solution (Masson Dye Set, BH0002, Wuhan Bolf Biotechnology Co., LTD., China) for a duration of 24 h. The following day, the sections were washed with tap water. Iron hematoxylin A and B were then mixed in equal proportion to form iron hematoxylin dye solution. Paraffin was then sliced into iron hematoxylin for 3 min, rinsed with tap water, differentiated with hydrochloric acid and alcohol for a few seconds, and rinsed with tap water once more. The paraffin sections were then placed into the ponceau acid fuchsin solution for 5**–**10 min, after which they were rinsed quickly with tap water. Treatment with phosphomolybdate aqueous solution was then carried out for 1**–**3 min. After phosphomolybdate, the samples were not washed, but instead directly dyed with aniline blue solution for 3**–**6 min. 1% glacial acetic acid (10000218, Sinopharm Group Chemical Reagents Co., LTD., China) was differentiated for a few seconds, and two cylinders of anhydrous ethanol were dehydrated. The paraffin sections should then be placed in anhydrous ethanol for 5 min, followed by n-butanol for a further 5 min, and finally xylene for a further 5 min. The sections should then be removed from the xylene and allowed to dry slightly before being sealed with neutral gum. Microscopy, image acquisition and analysis: collagen fibers, mucus, and cartilage are blue; muscle fibers, cellulose, and red blood cells are red.

**Section S3: Supplementary Figures and Tables**

**S3.1 The thermogravimetric analysis of the coral and CHA**

It is generally accepted that the thermal decomposition temperature range of calcium carbonate is 600**–**800°C, whereas that of HA is 800**–**1200°C. The results of the TGA show that the coral loses about 2%**–**3% of its mass when it is raised from room temperature to 300°C, mainly due to evaporation of adsorbed and structured water; when the temperature is raised from 300°C to 650°C, the mass is again reduced by about 2%, with a The mass loss from 300°C to 400°C may be due to the removal of lattice water, and the mass loss in 400**–**650°C may be due to the decomposition of organic components [11]. The significant mass loss in the 650**–**830°C range is mainly due to the decomposition of CaCO₃ into CaO and CO₂ after the coral material is heated to 650°C, and the mass loss starts to accelerate until the end of the thermal decomposition at 830°C, when the residual mass remains at about 54%. However, the trend of mass loss when CHA was raised from room temperature to 830°C was consistent with coral. The difference is that after exceeding 830°C to 1000°C, the remaining mass is about 70%. Thus, this further confirms that part of the mass loss of CHA when raised from room temperature to 830°C is due to thermal decomposition of the internally unconverted coral, and that the remaining mass of 70% is the sum of the masses of CaO and HA from the decomposition of CaCO₃. Therefore, in combination with the Figure 2B and the thermogravimetric graph, we inferred that Sr-CHA, Mg-CHA, and Sr-Mg-CHA might have formed a core-shell structure.


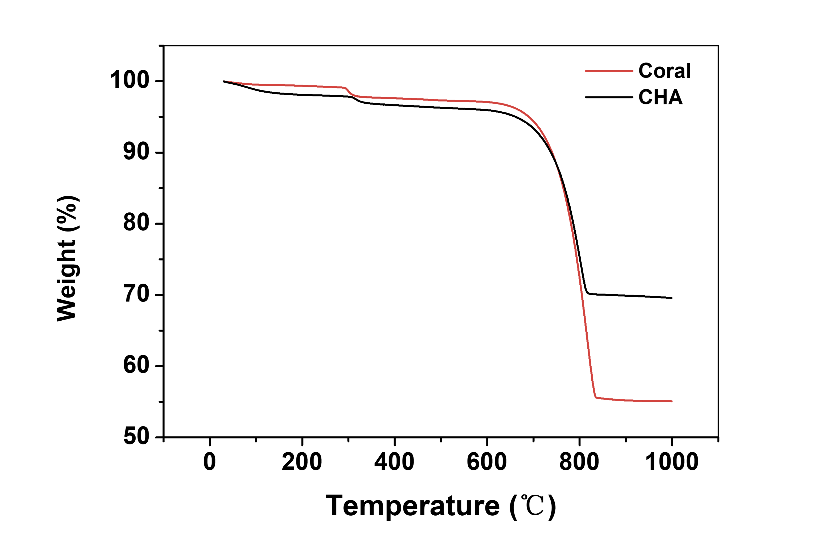


**Figure S1**. The thermogravimetric analysis of the coral and CHA

**S3.2 Semi-quantitative analysis of nanorod length in the Sr-CHA, Mg-CHA, and Sr-Mg-CHA structures.**


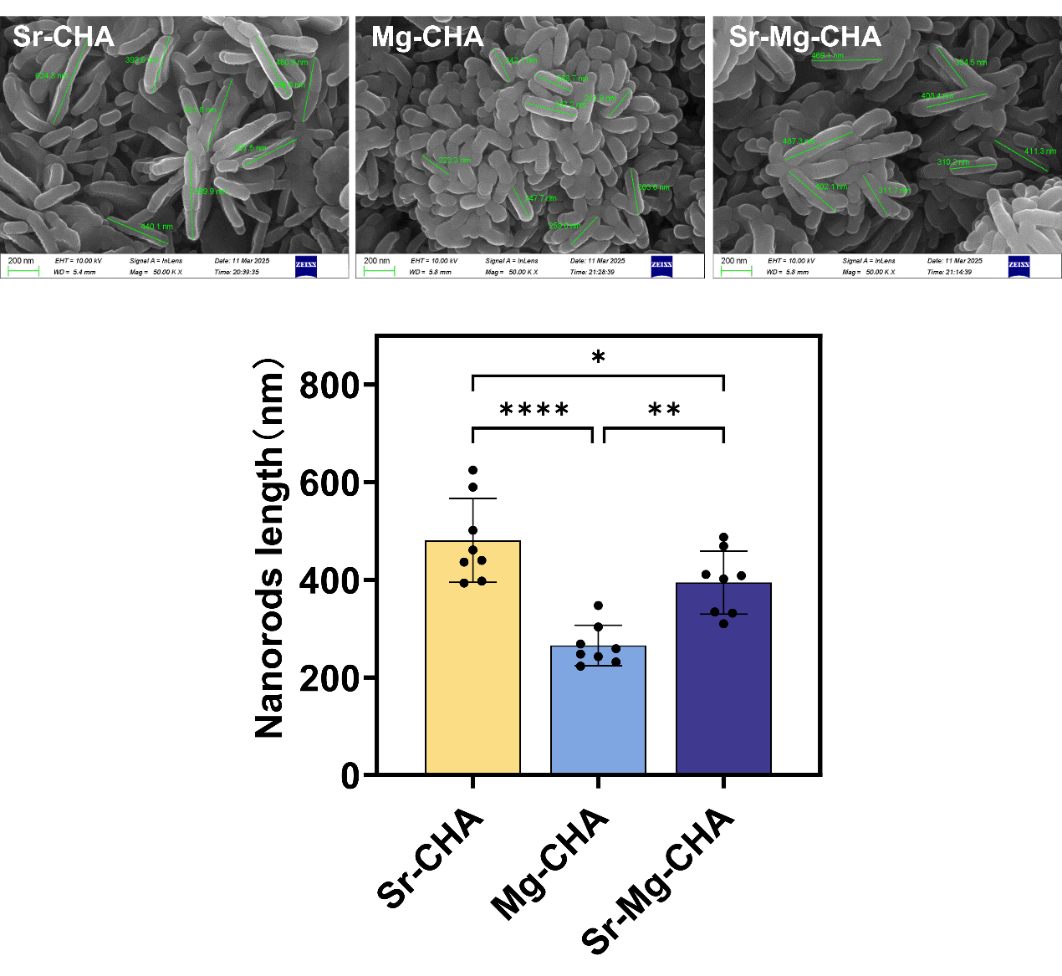


**Figure S2**. Semi-quantitative analysis of nanorod length in the Sr-CHA, Mg-CHA, and Sr-Mg-CHA structures.

**S3.3 Ratio of newly formed bone area in Masson-stained histological sections**

A semi-quantitative analysis of newly formed bone area ratio in Masson-stained histological sections was performed using ImageJ. At 4 weeks, the Sr-CHA, Mg-CHA, and Sr-Mg-CHA groups exhibited significant bone formation tendencies (*n* = 4, **p* < 0.05) compared to the Bio-Oss^®^ and Coral groups. By 8 weeks, the Sr-CHA and Mg-CHA groups demonstrated superior osteogenic effects than other groups (*n* = 4, ***p* < 0.01).


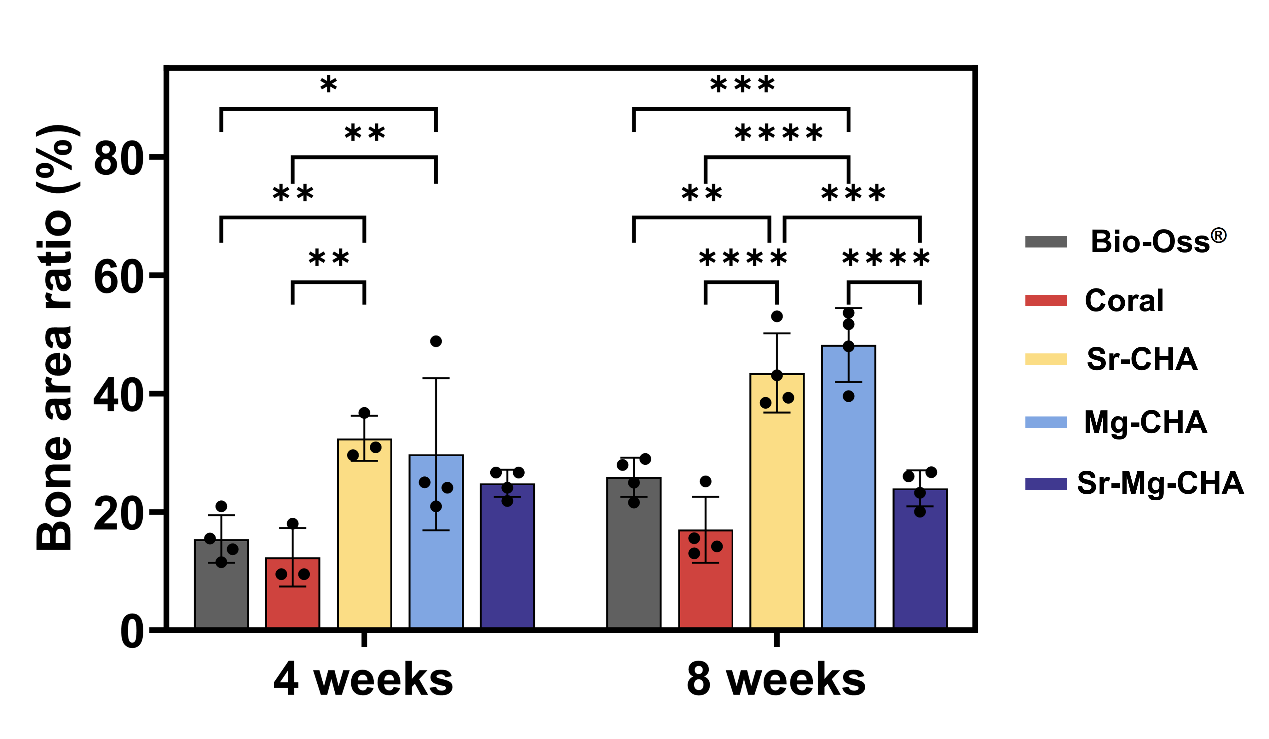


**Figure S3**. Ratio of newly formed bone area in Masson-stained histological sections

**References**

1. Yuan Q, Zhang Z, Yang Y, Jian Y, Li R, Dai X, Wu W, Zhong J, Chen C. Synthesis, characterization and biological performance study of Sr-doped hydroxyapatite/chitosan composite coatings. *Materials Chemistry and Physics* 2021;270:124752.

2. Li K, Li S, Ai F, Yan J, Zhou K. Fabrication and Characterization of Sr-doped Hydroxyapatite Porous Scaffold. *Jom* 2021;73:1745-1753.

3. Landi E, Tampieri A, Celotti G, Sprio S, Sandri M, Logroscino G. Sr-substituted hydroxyapatites for osteoporotic bone replacement. *Acta Biomater* 2007;3:961-9.

4. Ehret C, Aid-Launais R, Sagardoy T, Siadous R, Bareille R, Rey S, Pechev S, Etienne L, Kalisky J, de Mones E, Letourneur D, Amedee Vilamitjana J. Strontium-doped hydroxyapatite polysaccharide materials effect on ectopic bone formation. *PLoS One* 2017;12:e0184663.

5. Farzadi A, Bakhshi F, Solati-Hashjin M, Asadi-Eydivand M, Osman NAa. Magnesium incorporated hydroxyapatite: Synthesis and structural properties characterization. *Ceramics International* 2014;40:6021-6029.

6. Predoi D, Iconaru SL, Predoi MV, Stan GE, Buton N. Synthesis, Characterization, and Antimicrobial Activity of Magnesium-Doped Hydroxyapatite Suspensions. *Nanomaterials (Basel)* 2019;9.

7. Santos GG, Nunes VLC, Marinho S, Santos SRA, Rossi AM, Miguel FB. Biological behavior of magnesium-substituted hydroxyapatite during bone repair. *Braz J Biol* 2021;81:53-61.

8. Matić T, Zebić ML, Miletić V, Cvijović-Alagić I, Petrović R, Janaćković D, Veljović D. Sr,Mg co-doping of calcium hydroxyapatite: Hydrothermal synthesis, processing, characterization and possible application as dentin substitutes. *Ceramics International* 2022;48:11155-11165.

9. Jia B, Yang H, Zhang Z, Qu X, Jia X, Wu Q, Han Y, Zheng Y, Dai K. Biodegradable Zn-Sr alloy for bone regeneration in rat femoral condyle defect model: In vitro and in vivo studies. *Bioact Mater* 2021;6:1588-1604.

10. Cui X, Huang C, Chen Z, Zhang M, Liu C, Su K, Wang J, Li L, Wang R, Li B, Chen D, Ruan C, Wang D, Lu WW, Pan H. Hyaluronic acid facilitates bone repair effects of calcium phosphate cement by accelerating osteogenic expression. *Bioact Mater* 2021;6:3801-3811.

11. Shavandi A, Wilton V, Bekhit AE-DA. Synthesis of macro and micro porous hydroxyapatite (HA) structure from waste kina ( Evechinus chloroticus ) shells. *Journal of the Taiwan Institute of Chemical Engineers* 2016;65:437-443.
